# Phylogeography of the land snail genus *Orcula* (Orculidae, Stylommatophora) with emphasis on the Eastern Alpine taxa: speciation, hybridization and morphological variation

**DOI:** 10.1186/s12862-014-0223-y

**Published:** 2014-10-30

**Authors:** Josef Harl, Barna Páll-Gergely, Sandra Kirchner, Helmut Sattmann, Michael Duda, Luise Kruckenhauser, Elisabeth Haring

**Affiliations:** Central Research Laboratories, Museum of Natural History, Burgring 7, Vienna, 1010 Austria; Department of Integrative Zoology, University of Vienna, Althanstrasse 14, Vienna, 1090 Austria; Department of Biology, Shinshu University, Matsumoto, 390-8621 Japan; Department of Invertebrate Zoology, Museum of Natural History, Burgring 7, Vienna, 1010 Austria

**Keywords:** Integrative taxonomy, Biogeography, Speciation, Hybridization, Morphometric landmark analysis, Glacial refuges, Gastropoda

## Abstract

**Background:**

The Central and Southern European mountain ranges represent important biodiversity hotspots and show high levels of endemism. In the land snail genus *Orcula* Held, 1837 nine species are distributed in the Alps and a few taxa inhabit the Carpathians, the Dinarids and the Western Black Sea region. In order to elucidate the general patterns of temporal and geographic diversification, mitochondrial and nuclear markers were analyzed in all 13 *Orcula* species. We particularly aimed to clarify whether the Alpine taxa represent a monophyletic group and if the local species diversity is rather the result of isolation in geographically separated Pleistocene glacial refuges or earlier Tertiary and Quaternary palaeogeographic events. In order to test if patterns of molecular genetic and morphological differentiation were congruent and/or if hybridization had occurred, shell morphometric investigations were performed on the *Orcula* species endemic to the Alps.

**Results:**

The phylogenetic trees resulting from the analyses of both the mitochondrial (*COI*, *12S* and *16S*) and the nuclear (*H4/H3*) data sets revealed three main groups, which correspond to the three subgenera *Orcula, Illyriobanatica* and *Hausdorfia*. The reconstruction of the historic geographic ranges suggested that the genus originated in the Dinarides during the Middle Miocene and first colonized the Alps during the Late Miocene, giving rise to the most diverse subgenus *Orcula.* Within the latter subgenus (including all Alpine endemics) almost all species were differentiated by both molecular genetic markers and by shell morphometrics, except *O. gularis* and *O. pseudodolium*.

**Conclusions:**

The present study confirms the importance of the Alps as biodiversity hotspot and origin center of land snail diversity. The species diversity in the subgenus *Orcula* was likely promoted by Miocene to Pliocene palaeogeographic events and the insular distribution of preferred limestone areas. In some cases, speciation events could be linked to the divergence of populations in glacial refuges during the Pleistocene. Sporadic contact between geographically separated and reproductively not yet isolated populations led to intermixture of haplogroups within species and even hybridization and mitochondrial capture between species.

**Electronic supplementary material:**

The online version of this article (doi:10.1186/s12862-014-0223-y) contains supplementary material, which is available to authorized users.

## Background

European mountain ranges harbor a large number of endemic species and are generally considered as important biodiversity hotspots. Concerning diversity in European terrestrial snails, the IUCN Red List of Threatened Species lists 554 native species from rocky areas, 445 species from shrublands and 367 from forest habitats. Among the first category, rocky areas, the Dinarides represent the most diverse European mountain region with about two hundred native gastropods listed, followed by the Alps and the Carpathians, both with somewhat less than a hundred native species listed [1]. Obvious reasons favoring the diversity in mountain areas are the strong structuring of habitats with a wide range of ecological niches, and the availability of different geological substrates. As most land snails are calciphilous, mountain regions offering limestone bedrock are particularly rich in species and show high rates of endemism, whereas intermediate areas with siliciclastic bedrock constitute migration barriers for many taxa. The isolation in favorable habitats is therefore an important reason for diversification of Alpine land snails [2]. Moreover, the current distribution and diversity patterns of Central European land snails and other biota were affected most strongly by climatic events during the Pleistocene. The shifts in temperature and humidity, and the expansion of glaciers, resulted in the fragmentation of populations of many taxa, complete or local extinction, and the loss of genetic variation due to bottlenecks [3]. As large parts of the Alps were covered by glaciers during the Last Glacial Maximum (LGM; 30–18 kya [4]) and earlier glacial periods, their role as origin center of biodiversity was much discussed in the past decades. Reviews of early molecular genetic studies suggest that the Central European mountain ranges did not provide refuges during glacial maxima, but were settled recently from more southern regions [3,5,6]. However, more recent molecular genetic studies support the presence of northern refuges in the periphery of the Alps and in the Western Carpathians for particular organisms like plants and invertebrates [7-12]. Populations of former refuge areas were usually characterized by high genetic diversity and the presence of rare (private) alleles [13].

In the present study we investigate the phylogeny and phylogeography of the calciphilous land snail genus *Orcula* Held, 1837. *Orcula* species are high-spired snails, 5 to 10 mm in height, with internal lamellae extending to the aperture margins. The morphologies of these lamellae serve as the primary characters for species identification. Currently, 13 species are known, almost all distributed either in the Alps, the Carpathians or in the Dinarides. Only a single species, *Orcula zilchi* Urbański, 1960, was recorded from the western Black Sea region of Bulgaria and Western Anatolia. *Sphyradium dobrogicum* Grossu, 1986 was also classified within the genus *Orcula* in the Fauna Europaea checklist [14], but without any published reference. However, based on available information, the species was synonymized with *Sphyradium doliolum* (Bruguière, 1792) [15]. Data on type specimens and taxonomic considerations about all *Orcula* taxa are summarized in the type catalogue of [16].

So far, the most comprehensive investigation of the genus was performed by Gittenberger [17], who attempted to differentiate the Alpine *Orcula* taxa and the Dinaric *Orcula schmidtii* (Küster, 1843) by anatomical and shell morphological traits. Schileyko [18] also investigated the genital anatomy and formulated hypotheses about the relatedness of several species. Páll-Gergely et al. [15] were the first to study the anatomy of *Orcula jetschini* (Kimakowicz, 1883) and *O. zilchi* and, based on differences in the morphology of the penial caecum, the shell structure and the morphology of the aperture folds, subdivided the genus into three subgenera: (1) *Orcula*, (2) *Illyriobanatica* Páll-Gergely & Deli 2013 and (3) *Hausdorfia* Páll-Gergely & Irikov 2013. The subgenus *Orcula* includes all species distributed in the Alps, among them the type species *Orcula dolium* (Draparnaud, 1801), which has by far the widest distribution including the Alps, the Western Carpathians and surrounding lowlands [19]. In contrast, the Alpine endemics are almost exclusively restricted to rocky limestone habitats of the Northern and the Southern Calcareous Alps. Their distribution was mainly investigated by Zimmermann [20] and Klemm [2]. *Orcula gularis* (Rossmässler, 1837) shows a disjunct distribution in both the Northern Calcareous Alps (Salzburg, Styria and Upper Austria) and the Southern Calcareous Alps (Carinthia and East Tyrol). *Orcula austriaca* Zimmermann, 1932 shows a similar distribution but its main area is situated more easterly in the Northern Calcareous Alps of Lower Austria. *Orcula pseudodolium* Wagner, 1912 inhabits a few mountains in the Northern Calcareous Alps of Upper Austria only. A fourth species, *Orcula fuchsi* Zimmermann, 1931, is restricted to two mountains (Mt. Gippel and Mt. Göller) in the Northern Calcareous Alps of Lower Austria. The other four Alpine endemics are exclusively found in the Southern Calcareous Alps of Austria, Slovenia and Italy. Of these, *Orcula tolminensis* Wagner, 1912 stands out as it resembles conchologically a dwarf form of *O. gularis*, with similar aperture characteristics; it is known from three sites in Southern Carinthia and Slovenia only. *Orcula restituta* (Westerlund, 1887) is mainly distributed in the Slovenian Alps and *Orcula spoliata* (Rossmässler, 1837) has an isolated distribution about 200 km west in Trentino-Alto Adige (Italy). The fourth *Orcula* species of the Southern Calcareous Alps is *Orcula conica* (Draparnaud, 1801). It is common in the eastern part of the Southern Calcareous Alps, but was found at a single site in the Dinarides around the Plitvice lakes (Republic of Croatia) as well. The subgenus *Illyriobanatica* comprises *O. schmidtii*, *Orcula wagneri* Sturany, 1914 and *O. jetschini. O. schmidtii* and *O. wagneri* inhabit high mountain regions of the Dinarides, from the Republic of Serbia to southern Greece, and their distribution ranges do greatly overlap [21]. A delimitation of the two taxa is problematic, because the shell characters are highly variable and do not allow to clearly distinguish the two species (see pictures in [16] and [21]). Therefore, the latter two taxa are referred to as *O. wagneri*/*schmidtii* complex in the following. *O. jetschini* was reported from Romania and is the only *Orcula* species of the Western Romanian Carpathians. It is distributed in the Banat region, western Transylvania (including Crisana) and northern Oltenia. Its shell shows similarities to those of the Dinarid species *O. wagneri* and *O. schmidtii*, but it is a woodland species of lower elevations, occurring mainly among leaf-litter or decaying dead wood. *Orcula zilchi* represents the monotypic subgenus *Hausdorfia*. It is known from three localities in South-Eastern Bulgaria and from three sites in Western Anatolia only. Its habitat ranges from leaf litter of alluvial forests in the western Black Sea region to limestone rocks in Western Turkey [15,22].

The present paper addresses the evolutionary history of the genus *Orcula* in general as well as the phylogeographic patterns of the species endemic to the Alps in particular. We aimed to answer the following questions: Which geographic areas were inhabited by ancestral populations of *Orcula*? What are the causes for the high species diversity in the Alpine region? Is there a congruency between molecular genetic patterns and morphologically defined groups in the Alpine *Orcula* species? Are there indications for recent or past hybridizations between any of the species?

We performed the first phylogeographic study of the genus *Orcula* based on comprehensive mitochondrial (mt) and nuclear (nc) data sets including material of all 13 species. A molecular clock analysis was performed and combined with a phylogeographic range reconstruction to trace the distribution patterns of the mt lineages throughout time.

In order to test whether the differentiation in shell morphology is congruent with the molecular genetic groupings, morphometric landmark analyses were conducted with the group of *Orcula* species endemic to the Alps and the Alpine-Dinarid *O. conica*.

## Results

### Phylogenetic trees

**Figure 1:**
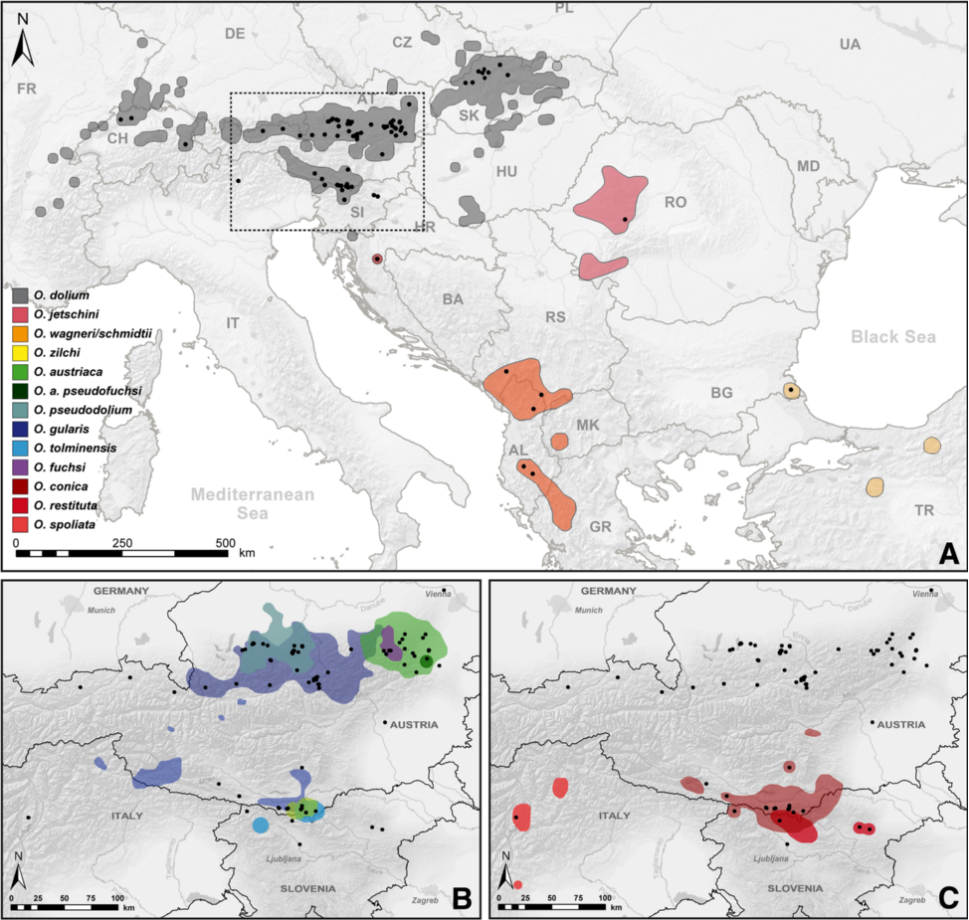
**Collection sites and distribution areas of** ***Orcula*** **species.** The three maps show the distributions of all *Orcula* species. Black dots mark the location of sample sites investigated during this study. **(A)** Distribution ranges *O. dolium, O. jetschini*, *O. wagneri* and *O. schmidtii*, and *O. zilchi*. The dotted line marks the area enlarged in Figures B and C. Map modified from [15]. **(B and** **C)** Distribution ranges *Orcula* species in the Alps.

**Figure 2:**
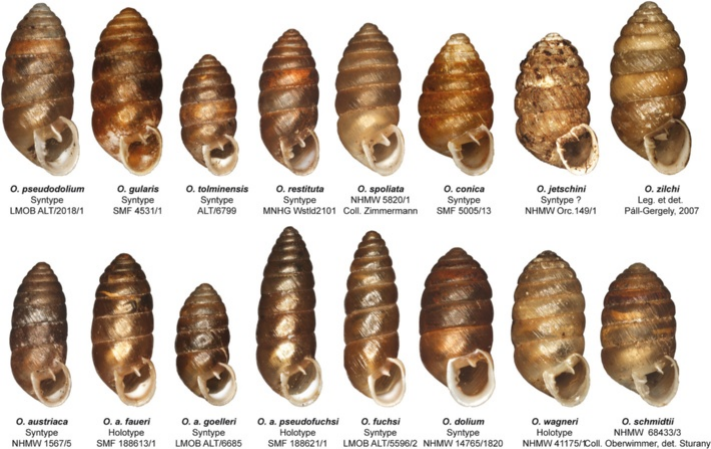
**Pictures of the** ***Orcula*** **taxa investigated.** The figure shows type specimens or selected individuals, respectively, of the 13 species investigated in the present study. Pictures of the three subspecies of *O. austriaca* are provided as well. Modified after Harl et al. [16].

A 655 bp fragment of the mitochondrial (mt) cytochrome c oxidase subunit I (*COI*) was analyzed in 295 specimens from 151 sites (Figure 1 and Table 1), including samples of all 13 extant *Orcula* species (Figure 2). The sequences of the *Orcula* species endemic to the Alps and the Alpine-Dinarid *O. conica* constituted three quarters of the samples. The nine species were in the focus of the study and we aimed to infer the degree of intraspecific molecular genetic (mtDNA) and morphological variation across the major parts of their distribution areas.

**Table 1:** **Sampling localities and individuals**

**Locality code**	**Locality**	**WGS84 (N)**	**WGS84 (E)**	**m asl**	**IndID**
**Western Romanian Carpathians**					
BAN1	RO, Bihor, Şuncuiuş, valley of Crisul Repede	45°56.405′	22°32.954′	200	**6646**, **6647** j
**Strandzha Mountains**					
STZ1	BG, Burgas, Strandzha Mts, Kondolovo vill.	42°6.144′	27°39.888′	330	**7021**, **7022**, **7023** z
**Dinarides**					
GJA1	AL, Kukës, Bicaj, Gjalica e Lumës	41°59.959′	20°27.680′	2060	**1393**, **1394** sch
MLK1	HR, Lika Senj, Plitvička Jezera NP, Galovac lake	44°52.616′	15°36.389′	573	3336, 3337, **3338** c
PRO1	ME, Podgorica, Rikavačko, summit area	42°34.483′	19°35.044′	1800	**3543**, **3544** w
PTK1	KO, Prizren, Paštrik, summit	42°13.204′	20°31.572′	1986	**3538** w
TOM1	AL, Berat, Tomor Mts, Tomor summit area	40°42.548′	20°8.662′	2200	**3541**, **3542** w
TOM2	AL, Berat, Tomor Mts, Maja e Ramiës	40°34.100′	20°15.522′	1850	**7138**, **7139** sch
**Northern Calcareous Alps**					
MRZ1	AT, NOE, Göller W-side, Gscheid	47°48.622′	15°27.084′	914	3024, 3025, 3026 a
MRZ2	AT, NOE, Göller S-side	47°46.941′	15°28.833′	1365	2955, **2956** a; 2951, **2952**, 2953 f
MRZ3	AT, NOE, Göller N-side, Turmmauer	47°48.705′	15°31.118′	812	3029, 3032, 3034 a; 3033, **3036**, 3037, 3038 f
MRZ4	AT, NOE, Göller N-side, Klopfermauer	47°48.705′	15°32.264′	741	2957, **2958**, 2959 a
MRZ5	AT, NOE, Göller N-side, Klopfermauer waterfall	47°48.705′	15°32.264′	741	2965, 2966, 2967, 2968 f
BGD4	AT, S, Saalfelden, Einsiedler	47°26.622′	12°51.720′	1029	**4119** d
DAC3	AT, OOE, Wiesberghaus, Wiesbergalm	47°31.529′	13°37.493′	1685	**1279** d
DAC4	AT, ST, Grimming, Grimming SE-side	47°31.172′	14°2.326′	1149	**5643** d; 5644, 5645 g
ENN3	AT, ST, Johnsbachtal, Langries-Brücke	47°33.681′	14°34.845′	631	5649, 5650, 5651 g
ENN4	AT, ST, Johnsbachtal, Kaderalpl	47°34.063′	14°34.899′	634	884, 886 g; **885** g*
ENN8	AT, ST, Johnsbachtal, Hellichter Stein	47°34.544′	14°35.348′	606	907, 908, 909 g
ENN9	AT, ST, Johnsbachtal, Gseng	47°34.085′	14°35.687′	766	5619, 5620, 5621 g
ENN10	AT, ST, Johnsbachtal, Upper Gseng	47°34.033′	14°36.106′	1039	5629, 5630, 5631 g
ENN11	AT, ST, Haindlkar, Zigeuner	47°35.114′	14°36.695′	603	898
ENN12	AT, ST, Ennstal, Haindlkar-Hütte 2	47°34.039′	14°36.773′	1078	5635, 5636, 5637 g
ENN20	AT, ST, Admonter Kalbling, Kalbling	47°32.678′	14°31.276′	1746	1354, 1355, 1356 g
ENN21	AT, ST, Johnsbachtal, Langries-bridge	47°33.681′	14°34.845′	667	375, **376** g; **377** g*
ENN22	AT, ST, Johnsbachtal, Langgrieß-estuary	47°33.666′	14°34.920′	652	631, 1937 g
ENN23	AT, ST, Johnsbachtal, Im Gseng 1	47°34.044′	14°34.934′	634	**924** g*
ENN24	AT, ST, Johnsbachtal, Im Gseng 2	47°34.266′	14°35.098′	613	899, 901 g
ENN25	AT, ST, Gstatterboden, Rauchbodenweg	47°35.493′	14°37.594′	587	612, 1130, 1353 g
ENN26	AT, ST, Johnsbachtal, Schneckengraben	47°31.782′	14°38.258′	1035	3090, 3091, 3092 g
ENN27	AT, ST, Johnsbachtal, Teufelsschlucht	47°31.648′	14°38.615′	969	3098, 3099, 3100 g
ENN28	AT, ST, Johnsbachtal, Teufelsklamm	47°31.843′	14°38.697′	1028	329, 330, **331** g
ENN29	AT, ST, Johnsbachtal, Ebner Mäuer	47°31.657′	14°38.818′	1061	3095, 3097 g
ENN30	AT, ST, Leobner, Leobner Mauer	47°29.861′	14°38.959′	2009	1350, 1351, **1352** g
ENN31	AT, ST, Hieflau, Wandaubruecke	47°37.715′	14°45.468′	488	3102, 3103, **3104** g
FOB1	AT, ST, Schöckl, Teufelstein	47°12.525′	15°27.665′	950	**858** d
FOB3	AT, NOE, Sonnwendstein, Almweg 1229	47°37.604′	15°51.141′	1229	**370** d
GUT1	AT, NOE, Tiefental, Ochbauer	47°52.638′	15°38.854′	739	**3042** d
GUT2	AT, NOE, Halbachtal, Rossbachklamm 1	47°54.327′	15°40.937′	649	**3070**, 3071, 3072 a
GUT3	AT, NOE, Halbachtal, Kleinzell	47°56.902′	15°42.874′	507	3044, 3045 a
GUT5	AT, NOE, Gösing, foot of the hill, W-side	47°44.431′	15°59.198′	700	**5476**, 5479 ap
GUT6	AT, NOE, Gösing, W-side	47°44.401′	15°59.205′	864	2938, **2939**, 2940, 2941 ap
GUT8	AT, NOE, Grafenberg, Seiser Toni	47°48.360′	16°0.503′	780	1115 a
GUT9	AT, NOE, Große Kanzel, Springlessteig 1	47°48.800′	16°0.600′	720	638, 639, 832, 2812, 2813 a
GUT10	AT, NOE, Große Kanzel, Johann-Stich ladder	47°48.649′	16°0.781′	820	598 a
GUT11	AT, NOE, Falkenstein, Herrengrotte	47°49.528′	15°42.674′	749	3065 a
GUT12	AT, NOE, Pernitz, Hirschwände	47°54.955′	15°56.297′	640	5660 a
KAI4	AT, T, Wilder Kaiser, Hochgrubach	47°33.357′	12°18.604′	1666	**1981** d
KWL1	AT, T, Pertisau, Achensee W-shore	47°27.423′	11°42.081′	955	**615** d
LEC1	AT, T, Imst, Hahntennjoch	47°17.225′	10°36.566′	1482	**5932** d
OOV1	AT, OOE, Traunstein, Naturfreunde-Steig	47°51.581′	13°49.056′	631	340, 341, 342 g
OOV3	AT, OOE, Traunstein, Mairalmsteig	47°51.960′	13°50.047′	1166	633, 634, 635 g
OOV4	AT, OOE, Traunstein, Naturfreunde-Steig (arch)	47°52.135′	13°49.591′	1482	359, 618 g
OOV5	AT, OOE, Hoher Nock, Feichtausee	47°47.550′	14°18.716′	1399	3360, 3361, 3362 p
OOV6	AT, OOE, Hoher Nock, Hauptkar 2	47°47.307′	14°18.953′	1733	3368, 3370, 3371 p
OOV7	AT, OOE, Hoher Nock, Feichtau (Seeweg)	47°47.783′	14°19.044′	1418	3364, 3365, 3366 p
OOV8	AT, OOE, Hoher Nock, Schneeberg	47°47.173′	14°19.048′	1877	3449, 3450, 3451 p
OOV9	AT, OOE, Hoher Nock, Feichtausee-Hütte 1	47°48.062′	14°19.144′	1360	2806, 2807, 2808 p
OOV10	AT, OOE, Hoher Nock, Feichtausee-Hütte 2	47°48.062′	14°19.144′	1360	3356, 3357, 3358, **3359** p
OOV11	AT, OOE, Hoher Nock, Haltersitz	47°47.466′	14°19.204′	1583	3352, 3353, 3354 p
OOV12	AT, OOE, Hoher Nock, Haltersitz 1	47°47.866′	14°19.260′	1388	3348, 3349, 3350 p
OOV13	AT, OOE, Hoher Nock, Eiseneck 1	47°48.755′	14°19.757′	1152	**3340**, 3341, 3342 p
OOV14	AT, OOE, Hoher Nock, Eiseneck 2	47°48.629′	14°20.182′	1298	**3344**, 3345, 3346 p
OOV15	AT, OOE, Krumme Steyrling-Tal, Kienberg	47°51.042′	14°20.308′	486	3453, 3454, 3455 p
OOV16	AT, OOE, Bodinggraben, Scheiblingau	47°47.883′	14°23.217′	580	5858, 5862, 5869, **5871** p
OOV17	AT, OOE, Stefflkogel, Grosser Weissenbach 2	47°50.967′	14°26.050′	440	**5827**, 5829, 5830 p
OOV18	AT, OOE, Stefflkogel, Grosser Weissenbach 1	47°51.050′	14°26.217′	420	**5819,** 5820**,** 5821 p
OOV19	AT, OOE, Stefflkogel, Unterer Zöbelgraben	47°50.750′	14°26.300′	550	**5838**, 5841 p
OOV20	AT, OOE, Stefflkogel, Oberer Zöbelgraben	47°50.633′	14°26.317′	720	5852, **5855** p
OSR3	AT, NOE, Seebenstein, Türkensturz	47°40.876′	16°8.277′	550	**5606** d
SNH21	AT, NOE, Fadensteig, Fadenwände 1525	47°47.253′	15°48.655′	1525	1359, 1361 a; **1362** d
SNH24	AT, NOE, Rax, Bismarcksteig	47°41.537′	15°42.426′	1787	323, **324**, 325, 327, 328, 830 a
SNH26	AT, NOE, Schneeberg, Hahnriegel	47°45.799′	15°49.928′	1804	6154, 6155, 6156 a
SZK8	AT, OOE, Hochschneid, Hochschneid	47°48.458′	13°41.533′	1624	**347** d
SZK10	AT, OOE, Feuerkogel, Pledialm	47°48.984′	13°43.549′	1444	1147 g
TEN2	AT, S, Hochthron, Thronleiter	47°29.310′	13°14.611′	1940	**1930** g
TOT1	AT, ST, Großer Priel, Vorderer Ackergraben	47°43.724′	14°2.504′	1027	3867, 3868, 3869 g
TOT3	AT, ST, Großer Priel, Welser-Hütte	47°43.493′	14°2.990′	1747	**3873** d; 3875, 3876, 3877 g
TOT4	AT, OOE, Grünau, Grünau S	47°51.867′	13°56.906′	530	6153 g
TOT5	AT, OOE, Grünau, Kasberg-Almtal	47°51.076′	14°0.365′	600	6151, **6152** g
WIW2	AT, NOE, St. Andrä-Wördern, Hagenbachklamm	48°18.660′	16°12.582′	191	**392** d
WIW4	AT, NOE, Steinwandgraben, Teufelsbrücke	47°56.711′	15°58.341′	672	5661, 5662 a
YBB7	AT, NOE, Lechnergraben, Talschluss	47°49.387′	15°2.673′	1160	89, 90, 91, 1113, 1114 g
**Southern Calcareous Alps**					
GAI1	AT, K, Kreuzen, Meierle	46°41.028′	13°26.015′	927	**1131** d
GAI4	AT, K, Dobratsch, Höhenrain	46°35.048′	13°41.041′	1900	**640** d
JUL4	SI, Dežela Kranjska, Triglav, Kluže	46°11.186′	14°25.474′	601	**1363** d
JUL5	SI, Dežela Kranjska, Vršič, N-side	46°27.558′	13°46.883′	960	1390, 1391 c
JUL6	SI, Dežela Kranjska, Trišič, Čadovlje	46°23.025′	14°19.776′	624	3473, **3474** r
KWN1	AT, K, Koschuta, Trögener Klamm	46°27.212′	14°29.811′	762	3899, 3900, 3901 c; **3905** d
KWN2	AT, K, Bärental, oberhalb Stausee	46°29.621′	14°9.708′	650	6142, 6143 g
KWN3	AT, K, Unteres Bärental, Bärental	46°29.227′	14°10.270′	770	**1938**, 1939 c
KWN4	AT, KR, Loibltal, Tscheppa gorge (Sapotnitza)	46°29.120′	14°15.810′	695	3469, 3470, **3471** c
KWN5	AT, KR, Loibltal, Tscheppa gorge	46°29.287′	14°16.680′	590	1525, 1526, 1527 c; 1950, 1951, 1952 g
KWN6	AT, ST, Großer Pyhrgas, Lugkogel	47°38.784′	14°22.431′	1424	3086, 3087, 3088 g
KWN7	AT, K, Hochobirmassiv, Freibachgraben 3	46°29.025′	14°26.049′	808	590, 591, **592** af
KWN8	AT, K, Kuhberg, Zell-Freibach	46°28.714′	14°26.092′	1001	3457,3458, 3459 af
KWN9	AT, K, Hochobir, Freibachgraben 2	46°49.877′	14°26.615′	788	4095, 4096, 4097 c
KWN10	AT, K, Hochobir, Freibachgraben 1	46°29.358′	14°26.748′	801	4091, 4092, 4093 af
KWN11	AT, K, Hochobir, Oberer Ebriachbach	46°30.630′	14°26.803′	900	3895, 3896, **3897** af; 3891, **3892**, 3893 t
KWN12	AT, K, Eisenkappel, Kupitzklamm	46°27.979′	14°36.915′	674	**4075**, 4076, 4077 t
STE1	SI, Dežela Kranjska, Solčava, Bela	46°19.906′	15°17.862′	751	1370, **1371**, 1379 r
STE2	SI, Dežela Kranjska, Solčava, Robanov Kot	46°18.682′	15°25.213′	1095	2824, 2825, **2826** r
VDN1	IT, Trentino, Sfruz, Sfruz-Credai	46°20.892′	11°8.387′	1070	5655, **5656**, **5657**, 5658 sp
**Western Alps**					
GLA1	CH, Sankt Gallen, Calfeisental, St. Martin	46°55.353′	9°21.333′	1347	**5934** d
SML4	CH, Bern, Moutier, Gorges de Court	47°15.360′	7°20.610′	650	**6144**, **6145** d
SML11	CH, Bern, Rumisberg, Schore	47°16.671′	7°38.210′	1066	**6140** d
**Western Carpathians**					
MLF1	SK, TN, Povazská Bystrica, Považský hrad N	49°8.734′	18°27.422′	500	**3938, 3939, 3942** d
MLF2	SK, TN, Povazská Bystrica, Manínska tiesňava S	49°8.398′	18°30.421′	380	**3919** d
MLF3	SK, TN, Povazská Bystrica, Manínska tiesňava N	49°8.366′	18°30.475′	400	**3915** d
MLF4	SK, ZI, Súľov-Hradná, Súľovské skaly	49°10.101′	18°34.633′	315	**3932** d
MLF6	SK, ZI, Rajecké Teplice, Skalky Strážovské S	49°8.115′	18°41.745′	470	**3947** d
MLF7	SK, ZI, Malá Fatra, Terchová-Vrata	49°14.664′	19°2.360′	564	**1372, 1373, 1376** d
STR1	SK, TN, Trenčianske Teplice, Malý Klepáč W	48°53.720′	18°10.649′	480	**3909** d
STR2	SK, TN, Strážovské vrchy, Valaska Beta	48°53.519′	18°22.469′	445	**1996** d
VEF1	SK, ZI, Ružomberok, Cebrat S-side	49°5.474′	19°17.174′	700	**3926** d
**OUTGROUP TAXA**					
**Dinarides**					
PIN1	GR, West Makedonia, Florina, Petres	40°44.066′	21°40.699′	580	**7002, 7003, 7004** *Orculella bulgarica*
**Northern Calcareous Alps**					
SNH23	AT, NOE, Semmering, Adlitzgraben	47°39.361′	15°50.168′	650	**833** *Sphyradium doliolum*
**Pontic Mountains (Turkey)**					
PON1	TR, Erzurum, Aşkale, Tercan tunnel	39°50.396′	40°33.984′	1880	**6606, 6607** *Orculella bulgarica*
PON2	TR, Erzurum, Aşkale, Aşkale	39°56.472′	40°36.395′	1652	**7105** *Orculella bulgarica*
**Baetic System (Spain)**					
BAE1	ES, Andalusia, Granada, Barrio los Parrales	37°18.437′	−3°14.807′	940	**7132** *Orculella aragonica*
BAE 2	ES, Andalusia, Granada, Barranco de las Ramillas	37°12,471′	−3°21.626′	1431	**7133** *Orculella aragonica*
BAE 3	ES, Andalusia, Granada, Cortijo del Olivar	37°31.952′	−2°44.382′	1431	**7135** *Orculella aragonica*
BAE 4	ES, Andalusia, Granada, La Torre spring	37°15.164′	−3°23,673′	1365	**7137** *Orculella aragonica*

**Abbreviations**

**Mountain range:** BAE, Baetic System; BAN, Banat; BGD, Berchtesgaden Alps; DAC, Dachstein Mts.; ENN, Ennstal Alps; FOB, Fischbach Alps; GAI, Gailtal Alps; GJA, Gjallica; GLA, Glarner Alps; GUT, Gutenstein Alps; JUL, Julian Alps; KAI, Kaiser Mts.; KWL, Karwendel; KWN, Karawanks; LEC, Lechtal Alps; MLF, Malá Fatra; MRZ, Mürzsteg Alps; OOV, Upper Austrian Prealps; OSR, Eastern Styrian Alps; PRO, Prokletje Mts.; PTK, Paštrik; SML, Swiss Plateau; SNH, Rax-Schneeberg Alps; STE, Steiner Alps; STR, Strážovské vrchy; STZ, Strandza Mts.; SZK, Salzkammergut Mts.; TEN, Tennengebirge; TOM, Tomor Mts.; TOT, Totes Gebirge; VDN, Valle di Non; VEF, Veľká Fatra; WIW, Wienerwald; YBB, Ybbstal Alps.

**Country names:** AL, Albania; AT, Austria; BG, Bulgaria; CH, Switzerland; DE, Germany; HR, Croatia; IT, Italy; KO, Kosovo; ME, Montenegro; RO, Romania; SI, Slovenia; SK, Slovakia.

**Federal districts:** BRN, Bern; BY, Bayern; FVG, Friuli-Venezia Giulia; K, Kärnten; KR, Kranjska; NOE, Niederösterreich; OOE, Oberösterreich; S, Salzburg; SG, St. Gallen; ST, Steiermark; T, Tirol; TN, Trenčiansky kraj; ZI, Žilinský kraj.

**List of localities and individuals included in the present study.** The first column indicates the geographic region. Each locality is defined by a unique locality code, representing a single collection site in one of the geographic (mountain) regions investigated. The locality column provides information on the respective localities. The GPS coordinates are given according to the World Geodetic System 1984 (WGS84) alongside the altitude in meters above sea level (asl). The individual IDs (IndIDs) of the specimens, together with information on the respective taxon names are provided for each locality. IndIDs in bold letters indicate that the complete marker set (*COI*, *12S*, *16S*, *H4*/*H3*) was sequenced in the respective specimens. Abbreviations of taxon names: a: *austriaca*, af: *austriaca faueri*, ap: *austriaca pseudofuchsi*, c: *conica*, d: *dolium*, f: *fuchsi*, g: *gularis*, g*: *gularis* (clade 2), j: *jetschini*, p: *pseudodolium*, r: *restituta*, sch: *schmidtii*, sp: *spoliata*, t: *tolminensis*, w: *wagneri* and z: *zilchi*.

In the Bayesian Inference (BI) and Maximum Likelihood (ML) trees calculated with the *COI* data set only, most species clades are well supported (data not shown), but the relationships between the clades are not resolved. The complete *COI* data of the seven Alpine endemics and *O. conica* are shown separately as phylogenetic networks.

In order to set up a reliable phylogenetic framework, we additionally analyzed sections of the mt *12S* ribosomal RNA (*12S*), *16S* ribosomal RNA (*16S*) and the nuclear (nc) histone *H3* and *H4* complex (*H4*/*H3*) in a selection of 87 individuals. We selected a representative sample of the major mt *COI* lineages and geographic distributions (including most type localities). Substitution saturation in the mt *COI* and the trimmed *12S* and *16S* alignments was examined with the test of Xia et al. [23], implemented in MEGA v.5.1 [24]. The alignments show only little substitution saturation, with I_ss.c_ values significantly larger than I_ss_ values (*P* = 0.000): *12S* (I_ss.c_ 0.699 > I_ss_ 0.218), *16S* (_Iss.c_ 0.722 > _Iss_ 0.249) and *COI* (I_ss.c_ 0.718 > I_ss_ 0.271). However, moderate substitution saturation (P = 0.0012) is observed in the 3rd codon positions of the *COI* with I_ss.c_ (0.686) and I_ss_ (0.581) values differing only marginally between each other.

**Figure 3:**
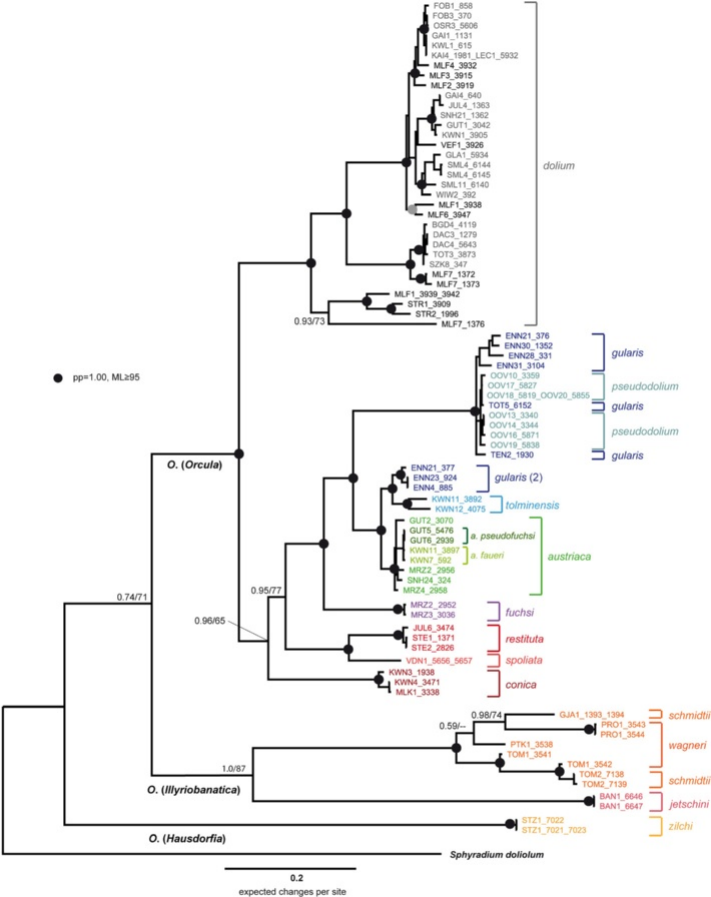
**BI tree of the concatenated mitochondrial sequences (** ***12S*** **,** ***16S*** **and** ***COI*** **).** Posterior probabilities and ML bootstrap values are provided for all nodes above species level. The scale bar indicates the expected number of substitutions per site according to the models of sequence evolution applied. The black and grey dots indicate nodes with high BI posterior probabilities and ML bootstrap values (see Figure). The colors of the specimen labels correspond to those used in Figures 4, 7 and 9.

**Figure 4:**
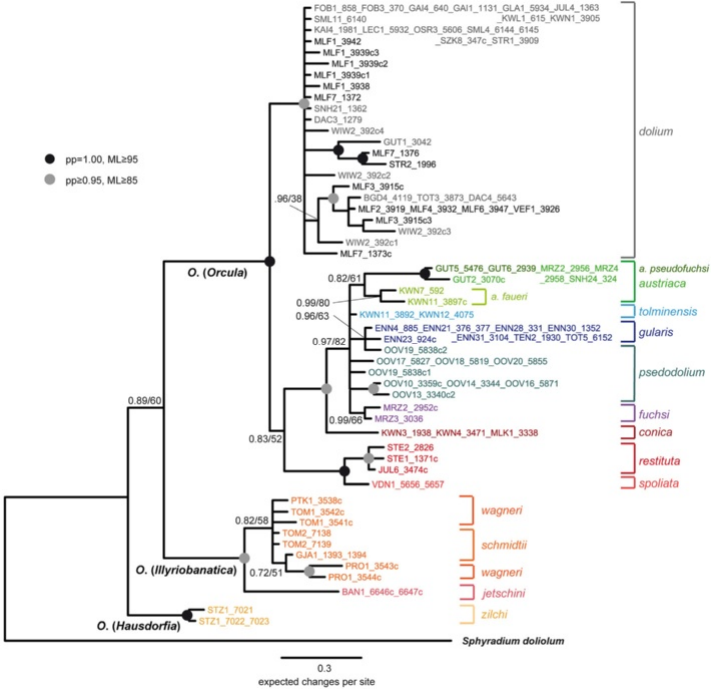
**BI tree of the nc** ***H4*** **/** ***H3*** **sequences.** Posterior probabilities and ML bootstrap values are provided for all nodes above species level. The scale bar indicates the expected number of substitutions per site according to the model of sequence evolution applied. The black and grey dots indicate nodes with high BI posterior probabilities and ML bootstrap values (see Figure). The colors of the specimen labels correspond to those used in Figures 3, 7 and 9.

The BI and ML phylograms calculated with the concatenated alignments (*COI*, *12S* and *16S*) show congruent, well resolved topologies with high support values for most of the nodes (Figure 3). The topology remains the same when the 3rd codon position of the *COI* is excluded from the data set, but most nodes show lower posterior probabilities or likelihood values, respectively (Additional file 1). The nc trees were calculated with the *H4/H3* data of the same subset of specimens. The overall topology with a division into three main clades representing the three subgenera *Orcula*, *Illyriobanatica* and *Hausdorfia* is illustrated by the nc tree as well (Figure 4). However, the nc data set is less variable, and support values for most nodes are lower in the nc tree:

The first main clade in the mt and nc trees corresponds to the subgenus *Orcula*, which comprises all *Orcula* species showing distributions in the Alps. *O. dolium* is clearly monophyletic and the sister group to a clade comprising the lineages of the Alpine endemics and the Alpine-Dinarid species *O. conica*. In the mt trees (Figure 3, Additional file 1), *O. conica* branches off from the basal node, whereas in the nc tree *O. spoliata* and *O. restituta*, the two being sister species, split basally (Figure 4). In the mt trees, the most basal nodes are weakly supported, while the relationships between the other Alpine endemics are well resolved: *O. fuchsi*, endemic to the Northern Calcareous Alps, is the sister group of a highly supported clade comprising *O. austriaca*, *O. gularis*, *O. pseudodolium* and *O. tolminensis*. Within this clade, *O. gularis* is paraphyletic because most specimens of *O. gularis* and *O. pseudodolium* show up in the same clade. Moreover, three specimens of *O. gularis* (IndIDs 377, 885 and 924) from three distinct sites (ENN4, ENN21 and ENN23; Styria, Austria) possess different mt haplotypes, being closely related to those of *O. tolminensis. O. austriaca* forms the sister clade of the latter two clades. Differing in a few substitutions or indels only, the *H4*/*H3* sequences do not allow resolving the relationships within the group of the latter species (Figure 4). However, this part of the *H4*/*H3* tree shows some peculiar patterns. There is a rather deep split between samples of *O. austriaca* from the Northern and the Southern Calcareous Alps, whereas these geographically isolated populations are barely differentiated in the mt sequences. There is also a larger diversity of nc haplotypes within *O. pseudodolium* when compared to the more widespread *O. gularis*.

The second main clade corresponds to the subgenus *Illyriobanatica* and includes *O. schmidtii* and *O. wagneri* from the Dinarides, and *O. jetschini* from the Western Romanian Carpathians. The sequences of *O. schmidtii* and *O. wagneri* form a highly supported clade in both the mt and the nc trees, but the two species are not monophyletic. Consequently, they are referred to as *O. schmidtii*/*wagneri* complex in the present study. *O. jetschini* is clearly separated from the *O. schmidtii*/*wagneri* clade, but the monophyly of the subgenus *Illyriobanatica* is well supported (Figures 3, 4). Apart from other patterns of sequence similarity, all taxa of the subgenus *Illyriobanatica* lack a section of approximately 230 bp in the non-coding *spacer* region of the *H4/H3* sequences.

A third major lineage is constituted by *O. zilchi*, representing the monotypic subgenus *Hausdorfia*. It inhabits the western Black Sea region and is not closely related to any other species in the trees. Similarly as the taxa of the subgenus *Illyriobanatica*, *O. zilchi* features a very short branch in the *H4*/*H3* tree (Figure 4), but not in the mt trees (Figure 3, Additional file 1).

### Molecular clock analysis and reconstruction of geographic range history

**Figure 5:**
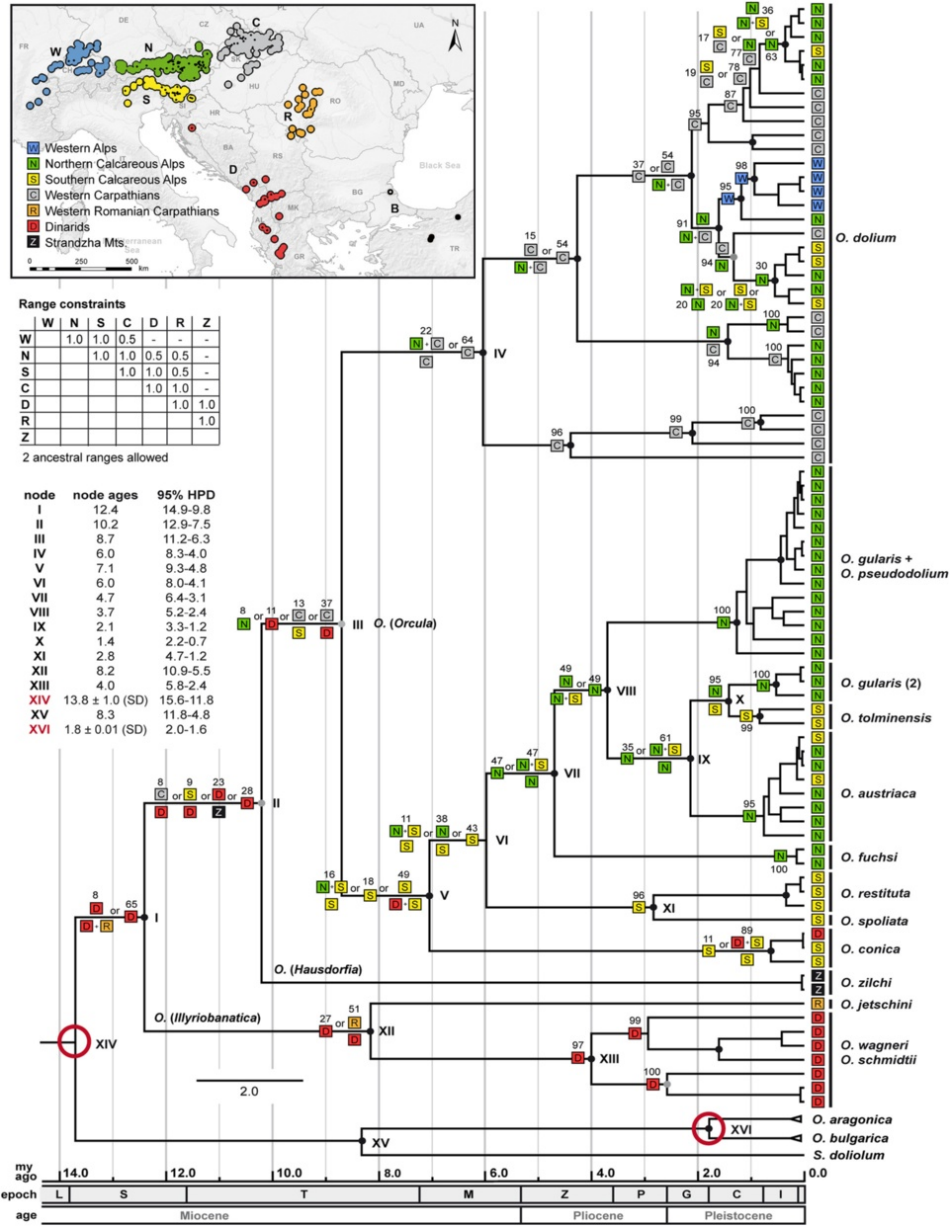
**Reconstruction of the historic geographic ranges.** The map shows the distribution areas of the genus *Orcula* in seven geographic areas: Western Alps, Northern Calcareous Alps, Southern Calcareous Alps, Western Carpathians, Western Romanian Carpathians, Dinarides and western Black Sea region. The sampling localities are indicated by black dots. The collection sites of the outgroup specimens *O. aragonica* in Spain (Baetic system) and *O. bulgarica* in Turkey (Anatolia) are not shown in the map. The linearized molecular clock dated maximum-clade-credibility tree shows the relationships of selected mt lineages (concatenated *16S, 12S* and *COI sequences*). Black dots indicate nodes with high posterior probabilities (see Figure). The colored symbols at the branch tips indicate the geographic origin of each haplotype. At the cladogenesis events (nodes), all alternative ancestral subdivision/inheritance scenarios with likelihoods of 10% or more are indicated, separated by an “or”, together with the respective likelihoods in%. When scenarios for cladogenesis events involve two ancestral areas, the symbol for the likely ancestral area/-s is/are provided left to each of the two branches. For nodes representing major splits, node ages and 95% posterior HPD intervals are indicated (see table). A time scale in mya is given below the tree. Abbreviations of the geological epochs: C: Chattian, A: Aquitanian, B: Burdigalian, L: Langhian, S: Serravallian, T: Tortonian, M: Messinian, Z: Zanclean, P: Piacenzian, G: Gelasian and C: Calabrian.

Molecular clock analyses and reconstructions of the geographic range histories were performed to analyze the temporal and geographic patterns of divergence of the mt lineages. The molecular clock dated trees were calculated in BEAST v1.7.5 [25] with the three mt markers (*COI*, *16S* and *12S*), using *Sphyradium doliolum*, *Orculella bulgarica* (Hesse, 1915) and *Orculella aragonica* (Westerlund, 1897) as outgroups. Since the inclusion of the sequences of the two *Orculella* species as additional outgroups affected the patterns in the *12S* and *16S* alignments, the resulting maximum clade credibility trees differ in their topology from the phylograms: The clade of the subgenus *Illyriobanatica* branches off from the basal node in the molecular clock trees (Figure 5, Additional files 2 and 3), whereas the subgenus *Hausdorfia* takes this position in the mt phylograms (Figure 3, Additional file 1). However, the node marking the first split within the genus *Orcula* obtained rather low support in all (also the nc histone) trees, and the relation between the three subgenera can still be considered as unresolved.

Three different approaches were performed to estimate the divergence times: (1) In the first approach (Additional file 2), the root of the tree (node XIV) was calibrated to the age of the fossil *Nordsieckula falkneri* (Hausdorf, 1995), the presumed most recent common ancestor of *Orcula*, *Sphyradium* and *Orculella*. The mean age of the node marking the split of *Illyriobanatica* from the subgenera *Orcula* and *Hausdorfia* (node I) was estimated to 12.2 mya (14.8 to 9.5 mya, 95% HPD interval). The split between the subgenera *Orcula* and *Hausdorfia* (node II) was estimated to 9.9 mya (12.6 to 7.1 mya, 95% HPD), and the date of divergence of *O. dolium* from the other eight Alpine species (node III) was estimated to 8.5 mya (10.9 to 6.0 mya, 95% HPD). (2) In the second approach (Additional file 3), the divergence date of the outgroup taxa *O. bulgarica* and *O. aragonica* (node XVI) was calibrated to the time of the first occurrence of ancestral *O. aragonica* in the fossil record of the Iberian Peninsula. The resulting estimated mean node ages were 13.4 mya (23.5 to 4.7 mya, 95% HPD) for node I, 11.0 mya (19.4 to 3.9 mya, 95% HPD) for node II, and 9.4 mya (16.6 to 3.4 mya, 95% HPD) for node III, respectively. (3) The third approach (Figure 5) was a combination of the first two and included the calibration of two nodes (XIV and XVI). The estimated mean node ages were 12.4 mya (14.9 to 9.8 mya, 95% HPD) for node I, 10.2 mya (12.9 to 7.5 mya, 95% HPD) for node II, and 8.7 mya (11.2 to 6.3 mya, 95% HPD) for node III, respectively.

The node ages are largely congruent in approaches 1 and 2, suggesting that the two different calibration points did not produce conflicting results. However, the resulting 95% HPD intervals are extremely large when only the split between the outgroup taxa *O. bulgarica* and *O. aragonica* (node XVI; approach 2) is calibrated (Additional file 3). In approach 3 (two node datings) (Figure 5), ranges are similar as in approach 1 (Additional file 2), indicating that the 95% HPD ranges are mainly influenced by placing a prior on the stem of the tree.

The reconstruction of geographic ranges was performed with Lagrange [26], using the linearized maximum clade credibility tree inferred with approach 3 (two dating points). The distribution area of the genus *Orcula* was classified into seven geographic mountain areas, and the ancestral lineages were allowed to occupy a maximum of two ranges at the same time: In the constrained model (1), migration was prohibited between very distant areas, because some ancestral ranges were biogeographically extremely unlikely, and different migration probabilities were assigned between adjacent and not immediately adjacent areas (Figure 5). In the unconstrained model (2), migration was permitted between all areas and with the same dispersal probabilities (Additional file 4).

The results are largely congruent at the outer branches of the trees, but the ancestral ranges and likelihoods estimated for the basal nodes/branches differ strongly between the two approaches. In the constrained model, the most recent common ancestor of the genus *Orcula* (node I) was likely distributed in the Dinarids (65%), and the ancestor of the subgenera *Orcula* and *Hausdorfia* (node II) was most likely distributed in the Dinarids (28%) or in an area additionally including the Bulgarian Strandzha Mts (23%) (Figure 5). The ancestor of the subgenus *Orcula* (node III) was most likely distributed in the Western Carpathians and the Dinarids (37%), the Western Carpathians and the Southern Calcareous Alps (13%), or in the Dinarids (11%). In the unconstrained model, a Dinarid ancestry of the genus is the most likely scenario as well, but with a lower probability (25%); an alternative range additionally includes the Northern Calcareous Alps (12%). Accordingly, the common ancestor of the subgenera *Orcula* and *Hausdorfia* (node II) was most likely distributed in either the Northern Calcareous Alps (16%) or in the Dinarids (11%). The unconstrained model also predicts different ranges for the common ancestor of the subgenus *Orcula* (node III): Northern Calcareous Alps (25%), Western Carpathians and the Southern Calcareous Alps (19%), or Western Carpathians and the Dinarids (15%).

The reconstruction of the geographic range history indicates that migrations to geographically distant mountain ranges represented rare events - the Western Black sea area, the Western Alps and the Western Romanian Carpathians were probably colonized only once. The Dinarides were probably re-colonized only once from the Southern Calcareous Alps during the Late Pleistocene or Holocene, namely by *O. conica.* However, in the subgenus *Orcula* migrations between Southern and Northern Calcareous Alps seem to have happened several times. Most complex patterns were found in *O. dolium*, which probably originated in the Western Carpathians and is now found in most limestone areas of the Alps. The results suggest that the species migrated repeatedly between the Western Carpathians and the Alps, and colonized the Western Alps probably only once.

### Genetic distances

In the mt data set, distances measured between the subgenera and species are extremely high (Table 2; Additional files 5 and 6). The uncorrected mean *p*-distances between the subgenera *Orcula* and *Illyriobanatica* are 28.6 (*12S*), 22.0 (*16S*) and 21.1% (*COI*), respectively. The mean distances between *Orcula* and *Hausdorfia* are 28.1 (*12S*), 25.2 (*16S*) and 24.7% (*COI*), whereas mean distances between *Illyriobanatica* and *Hausdorfia* are 31.4 (*12S*), 25.6 (*16S*) and 24.0% (*COI*), respectively. Surprisingly, the latter distances are even higher than the average distances between the genus *Orcula* and the outgroup *S. doliolum*, which are 30.9 (*12S*), 25.6 (*16S*) and 22.1% (*COI*). The species providing the largest intraspecific distances in the mt genes is *O. dolium* with 15.6 (*12S*)*,* 14.0 (*16S*) and 18.3% (*COI*), followed by the *O. wagneri*/*schmidtii* complex with 12.3 (*12S*), 11.6 (*16S*) and 15.5% (*COI*). Within the group of Alpine endemics, highest intraspecific distances are observed in *O. tolminensis* with 3.7 (*12S*), 4.8 (*16S*) and 4.9% (*COI*), in the clade comprising *O. gularis* and *O. pseudodolium* with 4.3 (*12S*), 3.9 (*16S*) and 4.6% (*COI*), and in *O. austriaca* with 1.5 (*12S*), 1.9 (*16S*) and 3.6% (*COI*), respectively. Haplotype and nucleotide diversities calculated for the separate species clades with the complete *COI* data set are high in all groups (Table 3). The sequence divergences are also high within the set of *H4*/*H3* sequences (Additional file 7). The mean *p*-distances are 4.3% (*H4*: 1.6; *H3*: 2.1; *spacer*: 8.5%) between *Orcula* and *Illyriobanatica*, and 3.7% (*H4*: 1.4; *H3*: 2.4; *spacer*: 7.2%) between *Orcula* and *Hausdorfia*. Contrary to the pattern in the combined mt trees, the branch lengths of *Illyriobanatica* and *Hausdorfia* are shorter in the tree calculated with the nc sequences (Figure 4), resulting in a considerably lower mean distance of 2.9% (*H4*: 1.6; *H3*: 2.1; *spacer*: 5.1%) between the two subgenera. Mean distances of the three *Orcula* subgenera towards the outgroup *S. doliolum* are 9.7% (*H4*: 2.8; *H3*: 6.4; *spacer*: 20.0%).

**Table 2:** **Primer sets**

**Region**	**Primer (5′ to 3′)**	**Origin**	**Fragment size**	**T°C RocheTaq**
***COI*** **fwd**	COIfolmerFw: GGTCAACAATCATAAAGATATTGG	[54]	655 bp	50°C
***COI*** **rev**	H2198-Alb: TATACTTCAGGATGACCAAAAAATC	[55]		
***12S*** **fwd**	*12S*GastFw: TTACCTTTTGCATAATGGTTAAACTA	[56]	669 - 725 bp	54°C
***12S*** **rev**	*12S*GastRv: CGGTCTGAACTCAGATCATG	[56]		
***16S*** **fwd**	*16S*LOrc1_fwd: TTACCTTTTGCATAATGGTTAAACTA	[19]	838 - 890 bp	54°C
	*16S*LOrc2_fwd: TTACCTTTTGCATAATGGTTAAATTA	[19]		
***16S*** **rev**	*16S*LOrc_rev: CGGTCTGAACTCAGATCATG	[19]		
***H4*** **/** ***H3*** **fwd**	Orc*H4*_left1: GTGCGTCCCTGGCGCTTCA	[19]	848 - 1095 bp	57°C
	Orc*H4*_left2: GGCGCTTCAGGGCGTACACT	[19]		
***H4*** **/** ***H3*** **rev**	Orc*H3*_right1: TGGGCATGATGGTGACACGCT	[56]		Finnzymes
				Phusion: 71°C
**intern fwd**	Orc*H4*S_left3: CGGTCTGAACTCAGATCATG	[19]		
**intern rev**	Orc*H3*S_right3: CGGTCTGAACTCAGATCATG	[19]		

Primer sequences for amplification and sequencing of the *COI*, *12S*, *16S* and *H4*/*H3* fragments. The *H4*/*H3* fragments were amplified with Finnzymes Phusion polymerase, wherefore the respective annealing temperatures are provided in addition.

**Table 3:** **Genetic diversity and** ***p*** **-distances in the** ***COI*** **data set**

**species**	**sequence no.**	**haplotype no. (h)**	**haplotype div. (Hd)**	**nucleotide div. (Pi)**	**mean dist.**	**max dist.**	**1**	**2**	**3**	**4**	**5**	**6**	**7**	**8**	**9**	**10**	**11**	**12**
***O. dolium***	35	33	0.997	0.107	10.4	18.3												
***O. gularis/pseudodolium***	129	74	0.974	0.028	2.9	5.7	20.2											
***O. gularis*** **(2)**	3	2	0.667	0.01	1.5	1.5	18.5	16.5										
***O. tolminensis***	6	5	0.933	0.031	3	4.9	19	16.1	5.5									
***O. austriaca***	56	26	0.959	0.017	1.6	3.8	17.6	16.6	6.2	7.9								
***O. fuchsi***	10	5	0.822	0.005	0.5	0.8	18.5	19.7	15.5	15.5	13.6							
***O. restituta***	8	3	0.75	0.003	0.4	0.6	16.9	20.1	15.2	13.8	13.7	15.2						
***O. spoliata***	4	1	-	-	-	-	18.4	18.8	16.1	15.5	15	16.6	10.1					
***O. conica***	19	4	0.509	0.011	1.2	2.3	18.3	19.9	16.4	16.1	16.1	17.2	14.3	14.9				
***O. schmidtii*** **/** ***wagneri***	9	7	0.944	0.107	11.3	14.8	21.1	22.5	20.8	20.5	19.1	20.8	18.6	19.9	18.9			
***O. jetschini***	2	1	-		-	-	21.6	23.2	22.6	22.1	21	24	19.8	20.3	19.4	20.1		
***O. zilchi***	3	2	0.067	0.099	0.2	0.2	24.9	23.4	26.1	25.5	26.4	26.6	21.7	21.8	23.6	24.2	22.3	
***S. doliolum***	35	33	0.997	0.107	-	-	21.2	19.8	19.4	19.4	19.4	22	18.3	18.8	20.1	21.9	21.6	24.2

### Mitochondrial diversity in the subgenus *Orcula*

**Figure 6:**
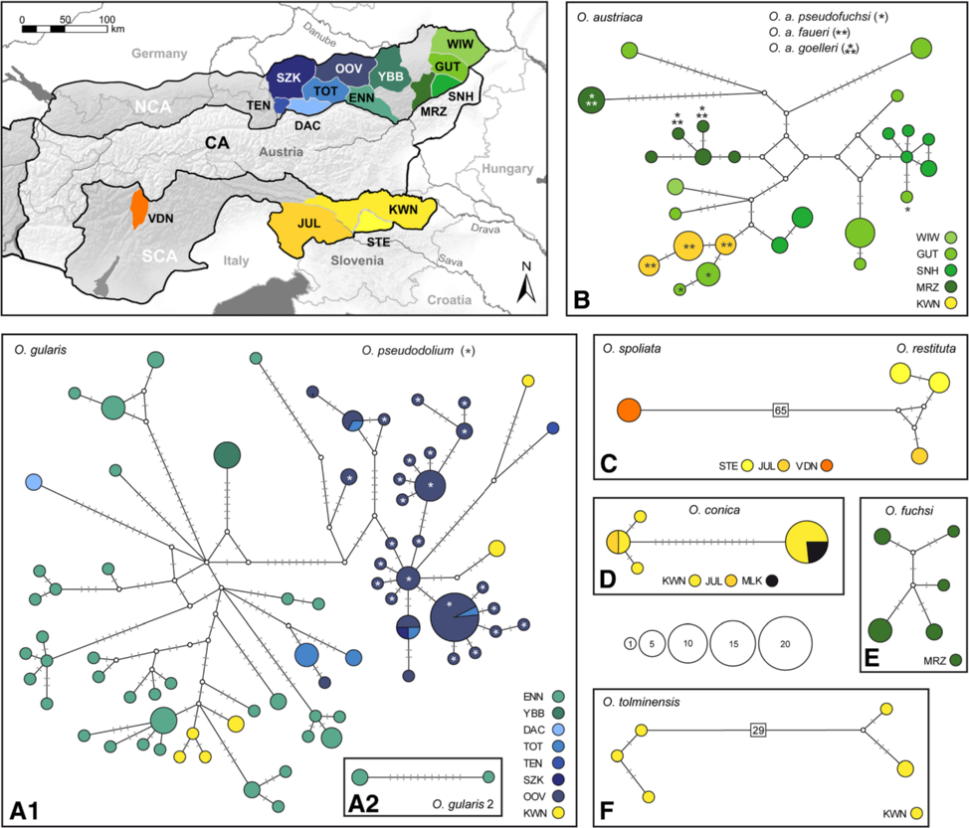
**Median Joining networks with** ***COI*** **sequences of the** ***Orcula*** **species endemic to the Alps.** The map shows the mountain regions inhabited by the species with colors corresponding to those used in the networks. The clades inferred from the phylogenetic tree analysis are shown in separated networks, numbered from **A–F**. The node colors correspond to the mountain regions shown on the map (except for the Dinarid Mala Kapela). The full names of the mountain regions are provided in the appendix of Table 1. The areas of the Northern (NCA) and the Southern Calcareous Alps (SCA) are shaded and encircled by black lines. The size of the circles in the networks corresponds to the number of sequences providing the same haplotype. Bars indicate the number of substitutions between nodes.

The *Orcula* species endemic to the Alps and the Alpine-Dinarid *O. conica* constituted more than three quarters of the samples analyzed and were in the focus of the present study. In order to display distributional patterns of the mt *COI* haplotypes, Median joining networks were calculated (Figure 6). The *COI* network corresponding to the *O. gularis*/*O. pseudodolium* clade in the mt trees (Figure 3, Additional file 1) comprises the majority of sequences (132) (Figure 6A1). Three specimens of *O. gularis* from the Northern Calcareous Alps (Ennstal Alps), clustering with *O. tolminensis* in the mt trees, are shown as a separate small network (Figure 6A2) because they are too diverged. Alternative scenarios explaining the relation of *O. gularis* and *O. pseudodolium* are discussed in detail in the following section. The main network of *O. gularis*/*O. pseudodolium* (Figure 6A1) is roughly divided into two clusters of haplotypes: the first includes samples of *O. gularis* from the Southern Calcareous Alps (Karawanks) and the Northern Calcareous Alps (Ennstal Alps, Dachstein, Ybbstal Alps and Totes Gebirge), and of a single specimen of *O. pseudodolium* (Northern Calcareous Alps: Upper Austrian Prealps). The second cluster includes all samples of *O. pseudodolium* (Northern Calcareous Alps: Upper Austrian Prealps), as well as a few haplotypes of *O. gularis* specimens from both the Northern (Salzkammergut Mts., Tennen Mts. and Totes Gebirge) and the Southern Calcareous Alps (Karawanks). The existence of Southern Calcareous Alpine haplotypes in both clusters indicates at least two independent migration events between the Southern Calcareous Alps and the Northern Calcareous Alpine populations. Since the Southern Calcareous Alpine haplotypes in the second cluster are highly derived, it can be assumed that *O. gularis* was already present in that area for a longer period, probably before the LGM (30–18 kya; [4]). However, the similarity of the Southern and Northern Calcareous Alpine haplotypes in the first cluster implies a second, recent migration event from the latter area to the Southern Calcareous Alps. A second clade, shown in a separate network, is formed by three specimens of *O. gularis* from the Northern Calcareous Alps (Ennstal Alps).

A further network (Figure 6B) comprises all 56 samples of *O. austriaca*, including sequences of the three subspecies *O. a. pseudofuchsi* Klemm, 1967, *O. a. faueri* Klemm, 1967 and *O. a. goelleri* Gittenberger, 1978. The haplotypes of *O. a. faueri* (Southern Calcareous Alps: Karawanks) are embedded within a larger variety of haplotypes of the Northern Calcareous Alpine populations of *O. austriaca*. They are separated only by a single mutational step from haplotypes present in the Gutenstein Alps. The haplotypes of *O. a pseudofuchsi* (Northern Calcareous Alps: Gutenstein Alps) are located in two different clusters of the network, one with haplotypes of the Southern Calcareous Alpine *O. a. faueri*, the second with haplotypes of a neighboring Northern Calcareous Alpine mountain area (Rax-Schneeberg). The haplotypes of *O. a. goelleri* (Mürzsteg Alps) also show up in two different clusters of the network. Concluding, none of the three subspecies of *O. austriaca* is clearly delimited from the nominate form in its mt *COI* sequences.

The networks representing *O. fuchsi* (Figure 6E) and the Southern Calcareous Alpine endemics *O. conica* (Figure 6D), *O. spoliata* and *O. restituta* (Figure 6C) are less complex. The sequences of the latter two species are shown in a combined network because we found only one haplotype in *O. spoliata*. Noticeable is that the Dinarid (Mala Kapela, Croatia) specimens of *O. conica* feature a haplotype which was found also in the Southern Calcareous Alps (Karawanks), more than 200 km south-east, indicating a recent long distance migration event. The network of *O. tolminensis* (Figure 6F) shows two highly diverged sequence clusters, separated by 29 mutational steps. The pattern might be the result of a long evolutionary history in the Southern Calcareous Alps.

### Morphological variation in the subgenus *Orcula*

**Figure 7:**
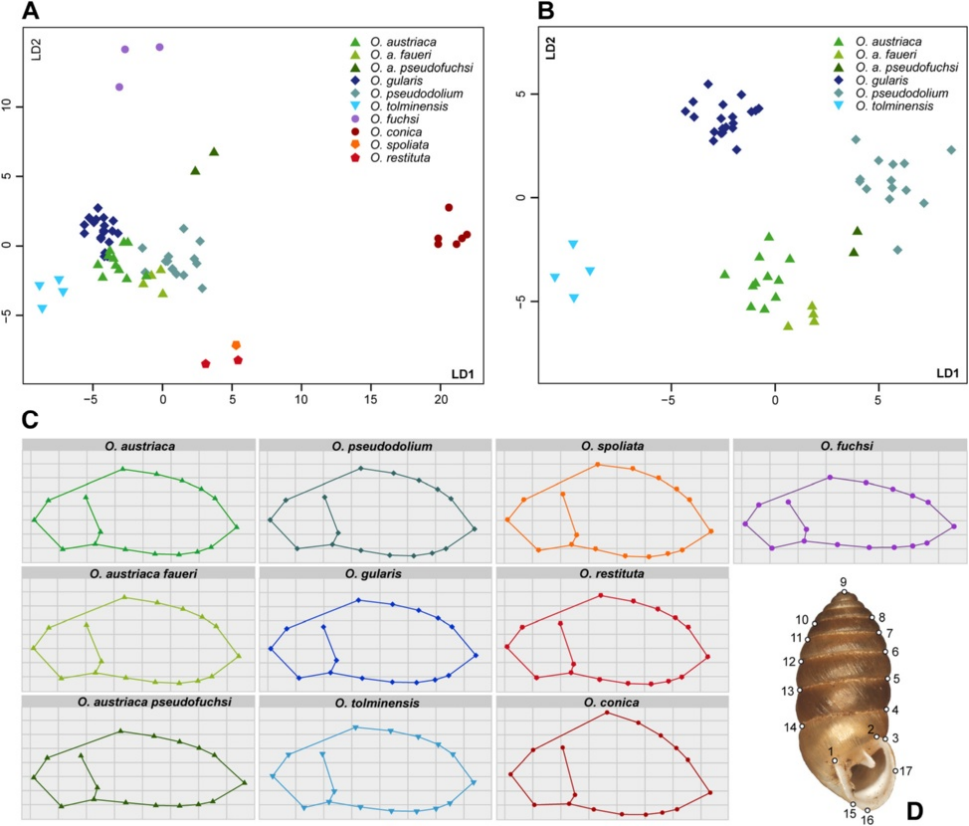
**Morphometric landmark plots.** **(A)** Linear discriminant analysis (LDA) with morphometric landmark data of all *Orcula* species endemic to the Alps. **(B)** Linear discriminant analysis with morphometric landmark data of the closely related *Orcula* species *O. austriaca* (including the subspecies *O. a. pseudofuchsi* and *O. a. faueri*), *O. gularis*, *O. pseudodolium* and *O. tolminensis*. Each symbol in the plot represents the consensus shape of conspecifics from the same locality. **(C)** Mean shapes calculated with all individuals of each taxon. The plots are corrected for size and therefore only reflect differences in the shell shape. The symbols represent the landmark points, set on the pictures of the shells photographed in frontal position. **(D)** Position of the morphometric landmarks on the projection of a shell in frontal position.

The morphometric analysis was performed with the landmark data of the Alpine *Orcula* species (except for *O. dolium*) to evaluate the amount of morphological differentiation between the species and subspecies, respectively. The high intraspecific variability of shell morphs, even from the same localities, complicates a clear separation of the different species based on data of single specimens only. However, a higher resolution is obtained when mean shapes of several specimens per locality are compared with each other (Figure 7). In the Linear Discriminant Analysis (LDA) including the mean shapes of all eight species, 85.02% of the total variance were explained by the first three discriminants (LD1: 59.37%, LD2: 16.09% and LD3: 9.56%; Figure 7A). In the LDA, shell shapes of *O. conica*, *O. fuchsi* and *O. tolminensis* are clearly differentiated. A distinct cluster is formed by *O. restituta* and *O. spoliata,* whose shells strongly resemble each other. The shell shapes of *O. a. pseudofuchsi* are unique and differ clearly from those of the nominate form *O. a. austriaca*, while *O. a. faueri* is hardly differentiated. The shape clusters of *O. gularis*, *O. austriaca* and *O. pseudodolium* are slightly overlapping (Figure 7A). However, when only the landmark data of the latter taxa (and the closely related *O. tolminensis*) are compared, the species form well-defined clusters (Figure 7B). In the respective LDA (Figure 7B), 88.79% of the total variance is explained by the first three discriminants (LD1: 42.33%, LD2: 28.38% and LD3: 18.08%).

## Discussion

### Phylogeny and phylogeography of the genus *Orcula*

One of the main aims of this study was to clarify the phylogenetic relationships among *Orcula* species and to test whether the Alpine *Orcula* species represented a monophyletic group. Based on a comprehensive phylogenetic data set including samples of all 13 *Orcula* species there is clear evidence that the nine species distributed in the Alps represent a monophyletic group corresponding to the subgenus *Orcula* as proposed by Páll-Gergely et al. [15]. Similarly, *Illyriobanatica* and *Hausdorfia* are each monophyletic in the mt and nc trees (Figures 3, 4). Previous considerations about the relationships of the *Orcula* species were made in particular by Gittenberger [17] and Schileyko [18], based on shell morphological and anatomical traits. Gittenberger’s [17] suggestion that *O. gularis*, *O. tolminensis*, *O. pseudodolium* and *O. austriaca* are close relatives, demarcated from species representing rather independent lineages (*O. dolium*, *O. spoliata*, *O. restituta*, *O. conica* and *O. fuchsi*), was confirmed in the present study. In contrast, the phylogenetic scheme proposed by Schileyko [18] is not consistent with our results and would result in paraphyletic species complexes.

The reconstruction of the geographic range history supports a scenario in which the genus *Orcula* originated in the Dinarides during the Middle Miocene (Figure 5). Thus, despite the fact that nine of 13 extant *Orcula* species are distributed in the Alps, the area was most likely not the center of origin, but was colonized from the Dinarids. The lineages of the three subgenera most likely split during the Middle and Late Miocene - during that time period, Alps, Dinarides and Carpathians were partly separated by a lateral branch of the Mediterranean Sea [27]. The separation of *O. dolium* from the group including the eight Alpine endemics (and the Alpine-Dinarid *O. conica*) was dated in the Late Miocene and could be explained with the formation of Lake Pannon, which separated Eastern Alps and Western Carpathians during the Tortonian and reached its maximum extent about 10 mya [28]. The results suggest that *O. dolium* originated in the Western Carpathians and colonized the Eastern Alps first during the Pliocene. The radiation into numerous mt lineages during the Pleistocene can be explained with divergence in separated glacial refuges as suggested by Harl et al. (2014) [19]. The diversification of the other *Orcula* species endemic to the Alps (including the Alpine-Dinarid *O. conica*) probably started shortly after the split from *O. dolium*, during the Late Miocene or the Lower Pliocene. Accordingly, the diversification of most Alpine *Orcula* species pre-dated the Pleistocene and their speciation cannot plausibly be explained solely by divergence in separate glacial refuges as proposed by Zimmermann (1932) [20] - only the split of the closely related *O. austriaca*, *O. tolminensis* and *O. gularis* is dated in the Pleistocene (Figure 5).

The species of the subgenus *Illyriobanatica* are distributed in the Dinarides and the Western Romanian Carpathians. Several other land snail taxa share similar distribution patterns and inhabit both mountain ranges, for instance the hygromiid *Xerocampylaea zelebori* (L. Pfeiffer, 1853), the aciculid *Platyla wilhelmi* (A. J. Wagner, 1910) and the clausiliid genus *Herilla* Adams & Adams, 1855 [29]. Our data suggest that the Dinarid *O. wagneri*/*schmidtii* complex and the Western Romanian *O. jetschini* split during the Late Miocene. The formation of Lake Pannon could have influenced their separation as well - parts of the Western Romanian Carpathians (including the current distribution area of *O. jetschini*) formed islands during the Middle Tortonian [28,30].

Summarizing, the reconstruction of the geographic range history indicates that the separation of the major groups within the genus *Orcula* was linked to palaeogeographic events which shaped Europe during the Miocene. The patchy distribution of limestone rock certainly constituted an important factor in the diversification of lineages because all *Orcula* species are more or less calciphilous. Since lowland areas like the Pannonian Basin featured almost no limestone rock, active migration between mountain ranges was probably hampered. The Pleistocene glaciations obviously had a strong impact on the current distributions of the Alpine endemics because their areas are all located near the eastern margins of the LGM glacier line. However, most of the latter species presumably diverged from each other in the Late Miocene and the Pliocene already, and not during the Pleistocene (Figure 5).

### Hybridization within the subgenus *Orcula*

**Figure 8:**
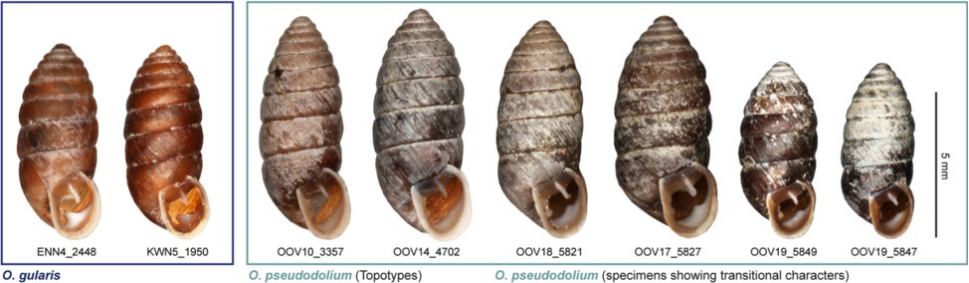
**Pictures of** ***O. gularis*** **and** ***O. pseudodolium*** **specimens.**

In most of the species of the subgenus *Orcula*, coherent mt and nc sequence patterns (Figures 3, 4, 6) as well as common morphological traits (Figure 7) were found. Thus, these species seem to be reproductively isolated from each other. Moreover, several species were found to occur sympatrically without indication for hybridizations: *O. austriaca* and *O. tolminensis*, *O. fuchsi* and *O. austriaca*, *O. gularis* and *O. conica*, *O. dolium* with *O. austriaca*, and *O. gularis* with *O. conica* and *O. fuchsi*. Nonetheless, our data strongly support that hybridization happened between *O. gularis* and *O. pseudodolium*. Although these two species could be discriminated by their shell forms in the morphometric analyses (Figures 7A and 7B) and provided different *H4/H3* sequences (except for a single specimen of *O. pseudodolium* from the potential hybridization area) (Figure 4), a clear assignment to one or the other species was not possible based on the mt sequences. Most mt haplotypes of *O. gularis* cluster with *O. pseudodolium* (Figures 3, 6), and only three single specimens of *O. gularis* (ENN21_377, ENN23_924 and ENN4_885) from the Ennstal Alps (Austria, Styria) show distinct mt variants, which form a sister clade of *O. tolminensis*. Specimens from four sites (OOV17, OOV18, OOV19, OOV20) even show transitional states in the expression of the palatal folds (from a backward orientated tooth to a diagonal bulge) (Figure 8), indicating recent hybridization.

Potential causes of non-monophyly of species in mt trees theoretically can be inferred from the depth of the coalescences in gene trees, geographical distribution of shared genetic markers, and concordance with results of admixture analyses of nuclear multilocus markers. [31]. In land snail species, incomplete lineage is discussed in species of the hygromiid genus *Xerocrassa* Monterosato, 1892 [32] and the helicid *Cornu aspersum* [33], whereas mt introgression is assumed to have happened in other *Xerocrassa* species [31] and in the camaenid genus *Euhadra* Pilsbry, 1890 [34].

The non-monophyly of *O. gularis* and *O. pseudodolium* in the mt trees might be explained by three different scenarios at least: (1) *O. gularis* acquired its mitochondria from *O. pseudodolium* by mt introgression, but genuine mt variants of *O. gularis* still exist in the population of the Ennstal Alps. (2) *O. pseudodolium* acquired its mitochondria from *O. gularis* by mt introgression, and the aberrant mt variants in the three *O. gularis* specimens of the Ennstal Alps were acquired from *O. tolminensis* by mt introgression. (3) The mixed mt patterns in the mt *O. gularis*/*O. pseudodolium* clade resulted from incomplete lineage sorting. Scenario 1 would require hybridization between *O. gularis* and *O. pseudodolium* only and, thus, provide a more parsimonious explanation than scenario 2. A close relationship between *O. austriaca*, *O. gularis* and *O. tolminensis* is also supported by similarities in the genital anatomy, whereas *O. pseudodolium* shows quite distinct traits [17]. Furthermore, *O. gularis* and *O. tolminensis* strongly resemble each other in their aperture traits by the unique presence of a palatal tooth. Incomplete lineage sorting (scenario 3) would not explain the relation between *O. pseudodolium* and *O. gularis* sufficiently. The mt sequence patterns in the network (Figure 6A) rather indicate that hybridization occurred at different points of time - some haplotypes of *O. gularis* are highly diverged from those of *O. pseudodolium* whereas others are identical or differ only by a few substitutions from each other. Moreover, the existence of the second *O. gularis* clade in the Ennstal Alps virtually cannot be explained by incomplete lineage sorting (Figure 3). Shedding more light on this topic would require additional sampling the potential hybridization area and analyzing (additional) nc markers from a larger number of specimens.

Another issue addressed in our study is the morphological variability within *O. austriaca*. Apart from the common form, three subspecies were described for *O. austriaca*. Among those, *Orcula a. pseudofuchsi* is of special interest because its shells are more elongated than those of the nominate form of *O. austriaca*. Klemm [35] hypothesized that *O. a. pseudofuchsi* represents an ‘intermediate’ between *O. a. austriaca* and *O. fuchsi*, or descended from the same common ancestor at least. Despite unequivocal shell morphological differences between specimens of *O. a. austriaca* and *O. a. pseudofuchsi* (Figure 7), the two taxa could not be delimitated by the mt and nc markers analyzed (Figures 3 and 4). Moreover, no intermediates between *O. austriaca* and *O. fuchsi* were found at Mt. Göller (Lower Austria) where both species co-occur and, in contrast to the assumption of Klemm [35], the two species were not even sister species (in the mt trees). Hence, the aberrant shell shape of *O. a. pseudofuchsi* is most likely not the result of hybridization but rather evolved uniquely in the population of *O. austriaca* from Mt. Gösing (Lower Austria). Similarly as in *O. austriaca*, conspecific populations strongly differing in shell morphology but not in their mtDNA were observed in the Western Carpathian populations of *O. dolium. Orcula dolium brancsikii* Clessin, 1887, exhibiting strongly elongated shells, was found next to populations with specimens featuring rather globulous shells, but a distinction of the two forms was not possible with the nc and mt markers used [19]. Another subspecies, *O. a. faueri*, inhabits the Southern Calcareous Alps, geographically separated from the nominate form of the Northern Calcareous Alps. Despite the geographic distance, the mt haplotypes of *O. a. faueri* are embedded within the diversity of the Northern Calcareous Alpine population, suggesting that *O. austriaca* colonized the Southern Calcareous Alps very recently (Figure 6). In contrast *O. a. faueri* could not be differentiated from the common form by its shell morphology (Figure 7). Only in the nc *H4/H3* trees there is a comparably strong bifurcation between the lineages of the Northern Calcareous Alps and the Southern Calcareous Alps, indicating that the evolutionary history of the species is probably more complex (Figure 4). A possible explanation could be that the populations of the Northern Calcareous Alps and the Southern Calcareous Alps diverged in allopatry, but intermixed recently, leading to mt capture and the loss of the genuine mt variants of *O. a. faueri*.

### Glacial refuges of the *Orcula* species endemic to the Alps

**Figure 9:**
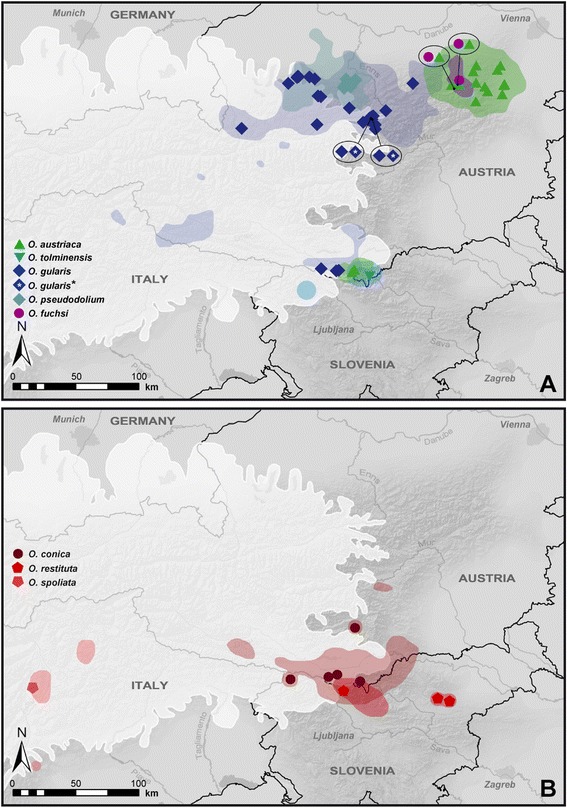
**Distribution of mt clades of the** ***Orcula*** **species endemic to the Alps.** As the distribution areas of five of the species overlap in the Southern Calcareous Alps, the data are displayed in two separate maps **(A and** **B)**. The colored symbols indicate the distribution of the mt clades, the color shaded areas represent the distribution areas of the respective *Orcula* species. In case that two species or species clades, respectively, were found at the same localities, the symbols are shown in ovals pointing towards the locality. The white shaded areas represent the glacier extent during the LGM (30–18 kya; [4]). The data on the maximum extent of glaciers during the LGM was published by [72], and modified by [8].

Although the diversification of the *Orcula* species endemic to the Alps probably started already in the Late Miocene and can only partly be attributed to speciation in glacial refuges, the Pleistocene LGM (30–18 kya; [4]) and earlier glacial maxima obviously affected the current distribution to a great extent. Since all of the latter species are strictly calciphilous, they probably could not survive the LGM in lowland areas surrounding the Alps like the closely related *O. dolium* [19]. However, most of the Alpine endemics show wide altitudinal ranges from the valleys up to high mountain areas (e.g., *O. gularis* from 400 to 2000 m asl) and are adapted to cold climates. Even though the LGM climatic snowline was 1000 to 1500 meters below the current level in peripheral ranges of the Eastern Alps [36], lower mountain ranges potentially provided suitable conditions during glacial periods. Moreover, the current distributions of all Alpine endemic *Orcula* species include areas not covered by ice during the LGM (Figure 9). The existence of Eastern Alpine refuges is also supported by several recent molecular genetic studies dealing with mountain plants [8,37,38] and invertebrates [9,10,39]. Besides, the Eastern Alpine margins harbor several endemic species with low active dispersal capabilities, among those blind troglobiotic beetles [40] and endemic land snail species restricted to high altitudes [41]. The genetic diversity within *Orcula* allows to conclude that the Alpine endemics outlasted the LGM and earlier Pleistocene cold stages probably in several smaller refuges at the periphery of both the Northern and the Southern Calcareous Alps and did not suffer from genetic bottlenecks.

## Conclusions

The results of the present study indicate that the evolutionary history of the genus *Orcula* dates back to the Middle Miocene. The three subgenera most likely derived from an ancestor that was distributed in the Dinarides, and their separation can be explained by palaeogeographic events preventing migration between the mountain ranges populated. Although the Alps were probably not the origin center of the genus, they gave birth to the majority of species. The structuring of the Alps, with two geographically separated major limestone areas (Southern and Northern Calcareous Alps), was of great importance for the diversification of the local *Orcula* species, most of them being strictly calciphilous. Within the group of Alpine endemics, most speciation events seem to predate the Pleistocene. Their current distribution patterns, however, were strongly shaped by the LGM glacier extent. Most taxa could be differentiated well by both morphologic and genetic traits, with the exception of *O. gularis* and *O. pseudodolium*. The latter two species differ in their shell morphology and nc DNA but cannot be distinguished by their mt DNA sequences, indicating mt introgression.

## Methods

### Study area and sampling

Samples of all 13 *Orcula* species were collected in the years 2007 to 2012 in ten different countries. Figure 1 shows the location of collection sites investigated. The distribution areas in Figure 1 are based on literature data [2,15,21,42], collection data of the Natural History Museum Vienna (NHMW) and the Senckenberg Museum (SMF), and data of the present study. Of a total of 115 localities investigated in the present study, most sites (101) were located in the Alps (Table 1). Elevation and position of the localities were determined via GPS. Samples were collected in various habitats covering an altitudinal range from 190 m to 2200 m above sea level (asl). One to four specimens of each species per site were prepared for the DNA sequence analyses and stored in 80% ethanol, following the protocol of Kruckenhauser et al. [43]. In addition, a number of empty shells were collected from the same sites for the morphometric analyses. DNA samples of the outgroup species *Orculella bulgarica* and *Orculella aragonica* were obtained from B. Gómez-Moliner (Universidad del País Vasco, Vitoria, Spain) and were already used by Arrébola et al. [44]. All other voucher specimens were deposited in the Natural History Museum Vienna (NHMW). In order to provide an overview of the taxa investigated, pictures of shells of selected type specimens are shown in Figure 2. The species and subspecies determination was based on a combination of shell characters (expression of aperture folds and shell form) and the geographic distributions reported in literature [2,21].

### Outgroup selection for phylogenetic trees and fossil calibration

**Figure 10:**
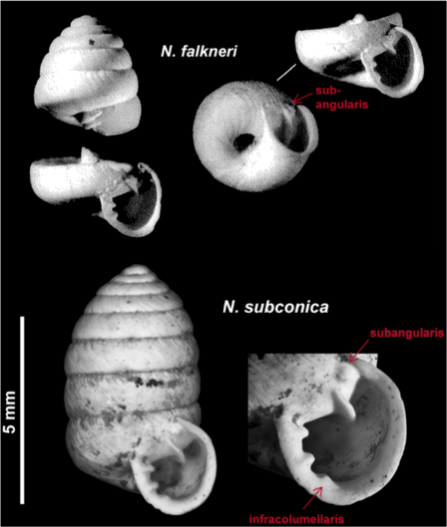
**Pictures of fossil orculid species** ***N*** **.** ***subconica*** **and** ***N. falkneri*** **.** *N. falkneri* from the type locality Nowa Wieś Królewska (Poland): BSP 1966 XXVI (holotype, left) and BSP 1952 XVIII/19 (paratype, right). Both specimens are deposited in the Bayerische Staatssammlung für Paläontologie und Geologie in Munich (Germany). The pictures were published in [48]. The specimen of *N. subconica* (Hochheim, Germany) is deposited in the Paleontological Department of the Natural History Museum Vienna (Austria): NHMW 1865/0011/0049.

In the course of the investigations on *Orcula*, samples of most other orculid genera (including *Alvariella* Hausdorf, 1996, *Orculella* Steenberg, 1925, *Pilorcula* Germain, 1912, *Pagodulina* Clessin, 1872, *Sphyradium* Charpentier, 1837 and *Schileykula* Gittenberger, 1983) were analyzed for the same set of mt and nc markers. Preliminary analyses based on this data set clearly support the monophyly of a group containing *Orcula*, *Schileykula*, *Sphyradium* and *Orculella*, with *Orcula* being the sister group to the other three genera (Harl et al. in prep.). Hence, all of these three genera represent equivalent outgroup taxa. We used the monotypic *Sphyradium doliolum* as outgroup for the calculation of the mt (*COI*, *12S*, *16S*) and nc (*H4/H3*) phylograms (Figures 3, 4). Within the family Orculidae, *S. doliolum* is by far the most widespread species with an area extending from Western Europe to Kyrgyzstan [45]. *Orculella bulgarica* and *Orculella aragonica* were additionally included as outgroups in the molecular clock analyses. *O. bulgarica* is the most widespread species within *Orculella*, distributed from southern Europe to Armenia [45], whereas *O. aragonica* is the only *Orculella* species of the Iberian Peninsula [46]. Their sister group relationship is supported by molecular genetic (*COI*, *16S*) and anatomical data [44], and a (within the genus uniquely) shared preference for wet habitats such as small marshes [46]. The paleontological record of *O. aragonica* comprises more than 30 Pliocene to Holocene sites, of which the Almenara-Casablanca karst complex (Castellón, Spain) features the oldest records (1.8 mya [47]). The site features a continuous Miocene to the Early Pleistocene fossil record, allowing to determine the species’ first occurrence in that area fairly precisely. One of the assumptions in the molecular clock analyses was that this first record coincides with the colonization of the Iberian Peninsula by ancestral *O. aragonica* or the split between *O. aragonica* and *O. bulgarica*, respectively. The second assumption was that the fossil orculid species *Nordsieckula falkneri* represents the most recent common ancestor of *Orcula*, *Orculella*, *Sphyradium* and *Schileykula* Gittenberger, 1983 (not included in the present study). The species is known from Middle Miocene sediments in Gründlkofen bei Landshut (Germany) and Nowa Wieś Królewska (Poland) and was previously classified into the genus *Orcula* [48]. Based on strong morphological similarities *N. falkneri* is considered as closely related to the genera *Orcula*, *Orculella*, *Sphyradium* and *Schileykula* [1], but as a distinctive trait it features a tooth-like subangularis [49], which was yet solely found in some of the extant North-African *Orculella* species. Its only congeneric *Nordsieckula subconica* (Sandberger, 1858) is known from Late Oligocene (Hochheim, Germany) to Early Miocene (Tuchořice, Czech Republic) successions [50]. *N. subconica* features an additional trait, a third columellar lamella (infracolumellaris), which is neither present in *N. falkneri* nor in any extant species of the genera *Orcula*, *Schileykula*, *Sphyradium* and *Orculella* (Figure 10). Since *N. falkneri* temporally succeeds *N. subconica* and was found in the same area, it might be a direct descendant of the latter. In the molecular clock analyses, the basal node of the tree was dated with the latest record of *N. falkneri* (Nowa Wieś Królewska, Poland), which was classified to the Astaracian (MN6/7) [51,52], corresponding to a time period comprising the Middle Miocene Langhian (15.97 to 13.65 mya) and the Serravallian (13.65 to 11.61 mya) [53]. The node age was set to 13.79 mya (SD ±1.0), the mean age between the lower boundary of the Langhian and the upper boundary of the Serravallian.

### PCR and sequencing

A total of 295 specimens were analyzed by means of molecular genetics (specimen numbers in brackets): *O. austriaca* (56), *O. conica* (19), *O. dolium* (35), *O. fuchsi* (11), *O. gularis* (84), *O. jetschini* (2), *O. pseudodolium* (48), *O. restituta* (8), *O. spoliata* (4), *O. tolminensis* (6), *O. wagneri/schmidtii* (9), *O. zilchi* (3), *S. doliolum* (1), *O. bulgarica* (6) and *O. aragonica* (4). Unique labels, consisting of a specimen number and a locality tag, were assigned to every specimen. The latter is a three letter code, with each code representing a geographic mountain region (Table 1). DNA was extracted using the QIAgen Blood and Tissue Kit. A section of the mt *COI* (655 bp) was analyzed in all specimens with the primers COIfolmerFw [12] (modified from Folmer et al. [54]) and H2198-Alb [55]. As the genetic distances between the *Orcula* species were extremely high, deeper splits in the phylogenetic trees could not be resolved with the *COI* alone. Therefore, additional mt and nc markers were analyzed in a selection of 86 individuals of *Orcula* and the eleven outgroup specimens: sections of the mt *12S* (673 to 725 bp) and *16S* (838–890 bp), and a sequence region comprising large parts of the nc histone genes *H3* (345 bp) and *H4* (259 bp), and the intermediate non-coding spacer (244–490 bp). The *12S* primers (*12S*GastFw and *12S*GastRv) and the *H4*/*H3* reverse-primer (Orc*H3*Right1) were published in [56]. The *16S* primers (*16S*LOrc1Fw, *16S*LOrc2Fw and *16S*LOrcRv) and the other *H4*/*H3* primers (Orc*H4*Left1, Orc*H4*Left2, Orc*H3*Sright3 and Orc*H4*SLeft3) were developed by [19]. For direct sequencing, DNA fragments were amplified with the RocheTaq^©^ DNA polymerase with 3 mM MgCl_2_. The PCR started with 3 min at 94°C, followed by 35 cycles with 30 s at 94°C, 30 s at the particular annealing temperatures (Table 3), 1 min at 72°C, and a final extension for 7 min at 72°C. Primer sequences and annealing temperatures are listed in Table 3. The *H4*/*H3* fragments were amplified with the primers Orc*H4*Left1 (or the alternative primer Orc*H4*Left2) and Orc*H3*Right1, but sequencing was performed with the internal primers Orc*H3*Sright3 and Orc*H4*SLeft3. Some specimens showed to be heterozygous regarding the non-coding spacer region. In those cases, the PCR was repeated with the proofreading Finnzymes Phusion^©^ polymerase. The PCR started with 30 s at 98°C, followed by 35 cycles with 10 s at 98°C, 10 s at the particular annealing temperatures (Table 3), 30 s at 72°C, and a final extension for 7 min at 72°C. The PCR products were excised from 1% agarose gels and purified using the QIAquick^©^ Gel Extraction Kit (QIAGEN), extended by A-endings with the DyNAzyme II^©^ DNA polymerase (Finnzymes) and then cloned with the TOPO-TA^©^ cloning kit (Invitrogen). Purification and sequencing (in both directions) was performed at LGC Genomics (Berlin, Germany) using the PCR primers, except for *H4*/*H3* (see above). The primer sequences are shown in Table 2. All sequences are deposited in GenBank under the accession numbers KM188500 - KM188950.

### Sequence statistics and phylogenetic tree reconstruction

The raw sequences were edited manually using Bioedit v.7.1.3 [57]. The alignment of the *COI* sequences was straightforward since there were no insertions or deletions (indels). Median-Joining networks were calculated for the eight Alpine endemics with Network v.4.6.0.0 (Fluxus Technology Ltd.) applying the default settings. In order to reduce unnecessary median vectors the networks were then post-processed with the MP (Maximum parsimony) option.

BI and ML phylograms were calculated based on the concatenated alignments (*COI*, *12S* and *16S*) of 86 *Orcula* specimens and the outgroup *S. doliolum*. The subset included all single specimens of *O. wagneri/schmidtii*, *O. jetschini*, *O. zilchi* and *O. dolium*, as well as a selection of specimens from the Alpine endemics. The *12S* and *16S* sequences were aligned with ClustalX v.2 [58] and adjusted manually. Less conserved sequence regions were excluded by trimming the alignment with TrimAl v.1.3 [59]. The original *12S* alignment contained 790 bp of which all 271 gap sites were removed using the ‘no gap’ option (removal of all sites containing gaps). Another 67 positions were excluded by applying the ‘strict’ option (trimming based on an automatically selected sequence similarity threshold [59]). The original *16S* alignment contained 945 positions of which 241 gap sites were excluded. Another 111 sites were removed with the ‘strict’ option. Subsequently, *COI*, *12S* and *16S* were concatenated and identical sequences were collapsed, resulting in a total of 86 unique haplotypes.

Substitution saturation was assessed for all single mt data sets using the test of [23], implemented in DAMBE v.5.2.78 [60]. In order to accommodate substitution saturation in the 3rd codon positions of the *COI* and to test the influence on the phylogeny, alternative trees were calculated excluding the third codon positions of the *COI*. Having collapsed identical sequences, the concatenated alignment of this data set contained a total of 77 haplotypes.

The optimal substitution models were determined for all individual data sets with JModelTest v.2.1.5 [61], based on the corrected Akaike Information Criterion (AICc). TrN + I + G was the best-fit substitution model for *COI*, *COI* ’1st and 2nd codon positions’ and *16S*, and TPM1uf + I + G for *12S*. The optimal model for the entire concatenated alignments was GTR + G + I. However, owing to the limited number of models applicable in MrBayes [62,63], the evolutionary model was set to GTR + G + I for all separate data partitions in the Bayesian analyses.

BI and ML phylograms were also calculated with the *H4*/*H3* data set. Similarly, the alignment was split into three partitions, namely *H4*, *spacer* and *H3*. The *spacer* was aligned with ClustalX v.2 [58] and all sites with gaps were removed. The data set contained 94 sequences (including additional clones when specimens were heterozygous for the *H4*/*H3*) of 87 specimens, which were collapsed to 53 unique haplotypes. Based on the AICc, the best fitting substitution models were K80 for *H3* (345 bp) and *H4* (259 bp), and K80 + G for the non-coding *spacer* (206 bp, gaps excluded). The optimal model for the concatenated *H4/H3* sequences was GTR + G.

The BI analyses were calculated using the concatenated (mt and nc) alignments, with three data partitions each, allowing MrBayes v.3.2.2 [62,63] to evaluate the model priors of each partition independently. Applying the respective model parameters, the analyses were run for 5x10^6^ generations each (2 runs each with 4 chains, one of which was heated), sampling every hundredth tree. Tracer v.1.5 [25] was used to assess whether the two runs had converged and when the stationary phase was reached, which was the case already after several thousand generations. In a conservative approach, the first 25% of trees were discarded as burnin and a 50% majority rule consensus tree was calculated from the remaining 37,500 trees.

ML bootstrap trees were calculated with MEGA v.5.1 [24], applying the models GTR + G + I to the mt and GTR + G to the nc sequences, respectively, but without using separate data partitions (since this option is not supported by MEGA v.5.1). For all data sets, 500 bootstrap replicates were performed using Subtree-Pruning-Regrafting (SPR) as heuristic method for tree inference.

Based on the alignment including all *COI* sequences, calculations of mean *p*-distances between species clades and maximum *p*-distances within the clades were performed with MEGA v.5.1 [24]. For the *12S* and *16S* alignments (gap sites excluded), mean *p*-distances between the species clades were calculated, and for the *H4*/*H3* alignments mean *p*-distances were calculated between the subgenera only. Haplotype and nucleotide diversities were evaluated with DnaSP v.5.10 [64] for the complete *COI* data set.

The sequence alignments used for the calculation of the mt and nc trees are provided in Additional files 8 and 9.

### Molecular clock analysis and reconstruction of geographic range history

The calculations of molecular clock dated linearized BI trees were performed in BEAST v.1.7.5 [25] with the concatenated mt sequences of *COI*, *12S* and *16S*. Apart from *S. doliolum* (1 specimen), 4 specimens of *O. bulgarica* and 6 specimens of *O. aragonica* were included as outgroup taxa, because these were used for dating the trees. The molecular clock analyses were calculated with the complete *COI* and the trimmed *12S* and *16S* alignments, following the same procedure as for the inference of the phylograms. Having removed all gap sites and performing the “strict” option in TrimAl v.1.3 [59], the *12S* and *16S* alignments contained 449 and 575 positions, respectively. For model selection and molecular clock analyses, identical sequences were collapsed to a total of 85 haplotypes (out of 97 specimens). The best fitting substitution models were calculated separately for each partition with JModeltest 2.1.5 [61], based on the AICc, resulting in the models HKY + G + I for *COI* and TN93 + G + I for*12S* and *16S*. The relative rate variation among lineages was tested separately for all three partitions with the molecular clock test implemented in MEGA v.5.1 [24] applying the optimal substitution models inferred with JModeltest 2.1.5. Since the null hypothesis of equal evolutionary rates throughout the trees were rejected at a 5% significance level (*P* =0), divergence times were estimated under a relaxed molecular-clock in all molecular clock analyses. The divergence times of the mitochondrial lineages were estimated using three different approaches: (1) The basal node of the tree was dated to 13.79 ± 1.0 (SD) mya (15.43 to 12.15 mya, 95% HPD interval), corresponding to the mean age between the lower boundary of the Langhian (15.97 mya) and the upper boundary of the Serravallian (11.61 mya) [53] or the Middle Miocene Astaracien, respectively, the period to which the fossils of *N. falkneri* were dated (see chapter ‘Outgroup selection for phylogenetic trees and fossil calibration’) (Additional file 2). (2) The node marking the split between *O. bulgarica* and *O. aragonica* was dated with the presumed first appearance of ancestral *O. aragonica* in the Iberian Peninsula around 1.8 mya as reported by [47]. The node age was set to 1.8 ± 0.1 (SD) mya (1.96 to 1.64 mya, 95% HPD interval) (Additional file 3). (3) A combination of approaches 1 and 2, applying the dating of nodes mentioned above (Figure 5).

In the sites settings of BEAUti v.1.7.5 (part of the BEAST package [25]), the best fitting substitution models were applied separately to each of the three partitions and the node datings were assigned in the prior settings. The speciation model Yule Process [65] was chosen as tree prior. The BEAST analyses were each performed with four independent runs for 10^7^ generations and every thousandth tree was sampled. Having checked whether the four runs had converged, these were combined with LogCombiner v.1.7.5 (part of the BEAST package). Subsequently, 25% of the trees were discarded as burnin and maximum clade credibility trees were calculated each from the remaining 30,000 trees.

The reconstruction of the geographic range history was performed in Lagrange [26], which uses a dispersal-extinction-cladogenesis (DEC) modeling for analyzing ML probabilities of rate transitions as a function of time. The rate-calibrated linearized tree from the molecular clock analysis (approach 3, two dating points) was prepared for Lagrange configurator [26], together with a range matrix in which each taxon/lineage was assigned to one of seven geographic regions, Northern Calcareous Alps (N), Southern Calcareous Alps (S), Western Alps (W), Western Carpathians (C), Dinarides (D), Western Romanian Carpathians (R) and Strandzha Mts. (Z). The maximum number of areas allowed for ancestors (= areas inhabited at the same time) was set to ‘2’. We tested two different models: (1) High migration probabilities (‘1.0’) were assigned to migration between directly adjacent areas, and lower probabilities (‘0.5’) were assigned to migration between not immediately adjacent areas (W to C; N to D; N to R). Migration was prohibited between geographically very distant areas (W to D; W to R; Z to W, N, S and C) (Figure 5). (2) Migration was permitted between all seven geographic regions with the same dispersal probabilities (‘1.0’) (Additional file 4).

The sequence alignments used for the calculation of the molecular clock tree are provided in Additional file 10.

### Morphological analyses

A total of 526 specimens were analyzed in the morphometric analyses. The sample included only specimens of the group of Alpine endemics: *O. austriaca* (66 specimens/12 localities), *O. a. faueri* Klemm, 1967 (60/4), *O. a. pseudofuchsi* Klemm, 1967 (21/2), *O. conica* (22/4), *O. fuchsi* (16/3), *O. gularis* (143/19), *O. pseudodolium* (141/14), *O. restituta* (18/2), *O. spoliata* (5/1) and *O. tolminensis* (34/4). Photographs of the shells were taken in frontal position with a WILD MAKROSKOP M420 and a NIKON DS Camera Control Unit DS-L2, all with the same magnification. Using tpsUtil v.1.44 [66], tps-files were created, then landmarks were set on 17 uniquely defined points of the shells’ projections in tpsDig v.2.12 [67]. Landmarks 1, 2 and 9 (Figure 7D) are type I, landmarks 16 and 17 type III, and all other points are type II landmarks as defined in [68]. Qualitative characters such as the expression of the aperture folds are not considered in the morphometric analyses. Since the shell forms differ quite among specimens of the same sample localities and, thus, increase the variability of the data set, consensus shapes (here: mean shapes of several specimens of a single locality) were created with tpsSuper v.1.14 [69]. Linear discriminant analyses (LDA) were performed with the landmark data of all Alpine endemics. Plots of the mean shapes were calculated with R v.3.2.0 [70] by using RStudio v.0.97.551 [71]. Morphometric analyses of *O. dolium* are not performed here, this topic will be discussed in detail in a forthcoming publication (Harl et al. in prep).

### Availability of supporting data

The data sets supporting the results of this article are included within the article and its supplementary files.

## Additional files

Additional file 1:
**BI tree of the concatenated mitochondrial sequences (** ***12S*** **,** ***16S*** **and** ***COI*** **1st2nd positions).** Posterior probabilities and ML bootstrap values are provided for all nodes above species level. The scale bar indicates the expected number of substitutions per site according to the models of sequence evolution applied. The black dots indicate nodes with high BI posterior probabilities (1.0) and ML bootstrap values (≥95). The colors of the species clades/labels correspond to those used in Figures 4, 7 and 9. Additional file 2:
**Linearized molecular clock dated tree (approach 1).** Maximum-clade–credibility tree calculated with BEAST, using the concatenated alignments of *12S*, *16S* and *COI*. The root of the tree (node XIV) was calibrated to the age of the fossil *Nordsieckula falkneri*, the presumed most recent common ancestor of *Orcula*, *Sphyradium* and *Orculella*. Node bars indicate the 95% HPD ranges estimated for each node. Mean node ages and 95% HPD ranges are provided for the major splits. A time scale in mya is given below the tree. Abbreviations of the geological epochs: C: Chattian, A: Aquitanian, B: Burdigalian, L: Langhian, S: Serravallian, T: Tortonian, M: Messinian, Z: Zanclean, P: Piacenzian, G: Gelasian and C: Calabrian. Additional file 3:
**Linearized molecular clock dated tree (approach 1)** **.** Maximum-clade–credibility tree calculated with BEAST, using the concatenated alignments of *12S*, *16S* and *COI*. The divergence date of the outgroups *O. bulgarica* and *O. aragonica* (node XVI) was calibrated to the time of the first occurrence of ancestral *O. aragonica* in the fossil record. Node bars indicate the 95% HPD ranges estimated for each node. Mean node ages and 95% HPD ranges are provided for the major splits. A time scale in mya is given below the tree. Abbreviations of the geological epochs: C: Chattian, A: Aquitanian, B: Burdigalian, L: Langhian, S: Serravallian, T: Tortonian, M: Messinian, Z: Zanclean, P: Piacenzian, G: Gelasian and C: Calabrian. Additional file 4:
**Reconstruction of the historic geographic ranges (unconstrained model).** The linearized molecular clock dated maximum-clade-credibility tree shows the relationships of selected mt lineages (concatenated *16S, 12S* and *COI sequences*). Migration was permitted between all areas and with the same dispersal probabilities. Black dots indicate nodes with high posterior probabilities. The colored symbols at the branch tips indicate the geographic origin of each haplotype. At the cladogenesis events (nodes), all alternative ancestral subdivision/inheritance scenarios with likelihoods of 10% or more are indicated, separated by an “or”, together with the respective likelihoods in%. When scenarios for cladogenesis events involve two ancestral areas, the symbol for the likely ancestral area/-s is/are provided left to each of the two branches. For nodes representing major splits, node ages and 95% posterior HPD intervals are indicated (see Table). A time scale in mya is given below the tree. Abbreviations of the geological epochs: C: Chattian, A: Aquitanian, B: Burdigalian, L: Langhian, S: Serravallian, T: Tortonian, M: Messinian, Z: Zanclean, P: Piacenzian, G: Gelasian and C: Calabrian. Additional file 5:
**Mean and maximum genetic** ***p*** **-distances in the** ***12S*** **data set.**
Additional file 6:
**Mean and maximum genetic** ***p*** **-distances in the** ***16S*** **data set.**
Additional file 7:
**Mean and maximum genetic** ***p*** **-distances in the** ***H4*** **/** ***H3*** **data set.**
Additional file 8:
**Sequence alignments (** ***COI*** **,** ***12S*** **,** ***16S*** **) used for the reconstruction of the combined mt tree (Figure** 3 **).**
Additional file 9:
**Sequence alignment used for the reconstruction of the nc** ***H4/H3*** **tree (Figure** **4** **).**
Additional file 10:
**Sequence alignments (** ***COI*** **,** ***12S*** **,** ***16S*** **) used for the reconstruction of the molecular clock tree (Figure** 5 **).**
