# Supplementary figures and images for: Phylogeography of the land snail genus Orcula (Orculidae, Stylommatophora) with emphasis on the Eastern Alpine taxa: speciation, hybridization and morphological variation
Source: BMC Evol Biol. 2014 Oct 30;14:223. doi: 10.1186/s12862-014-0223-y (PMC4219030; doi:10.1186/s12862-014-0223-y)

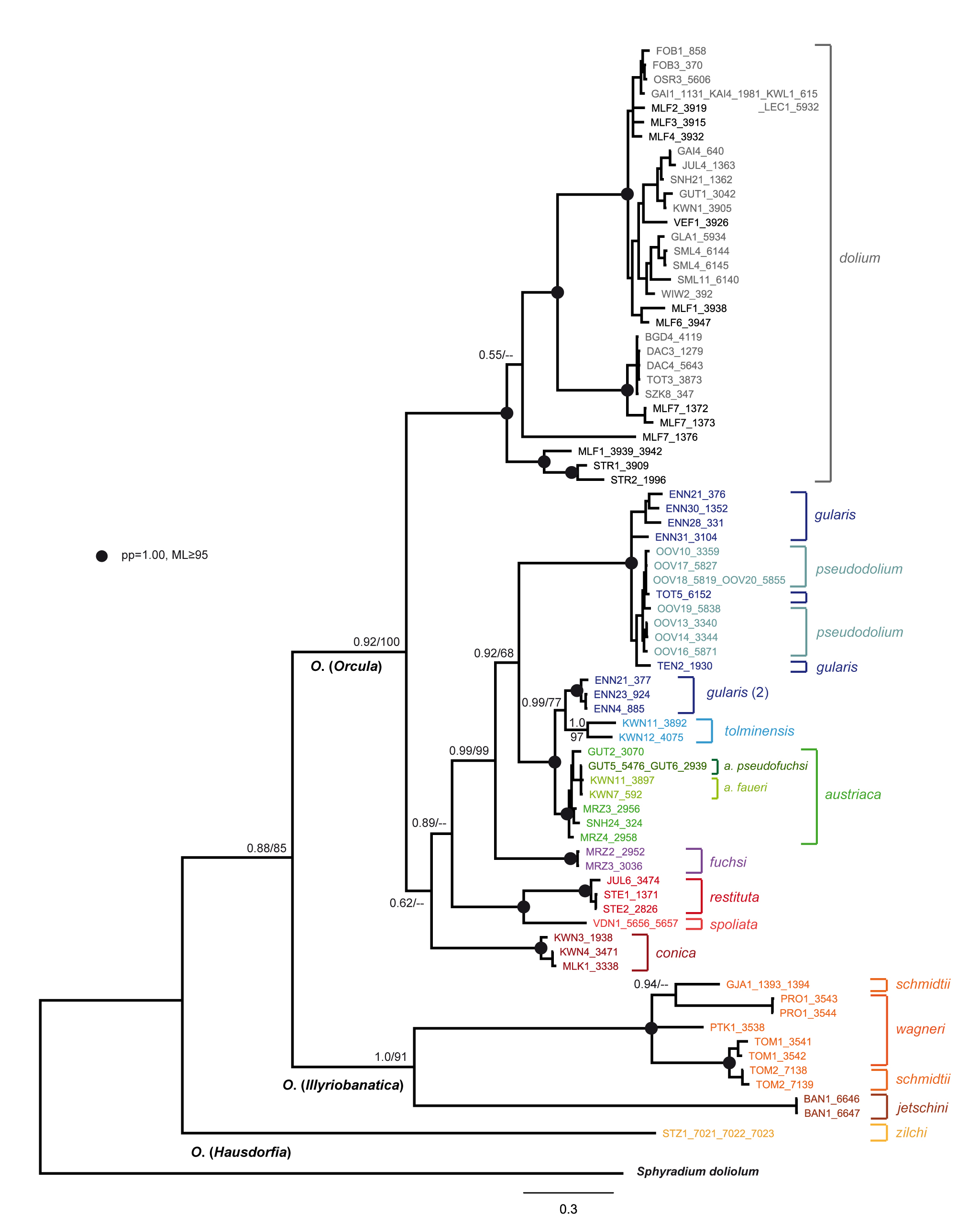

Supplement: Additional file 1: — BI tree of the concatenated mitochondrial sequences ( 12S , 16S and COI 1st2nd positions). Posterior probabilities and ML bootstrap values are provided for all nodes above species level. The scale bar indicates the expected number of substitutions per site according to the models of sequence evolution applied. The black dots indicate nodes with high BI posterior probabilities (1.0) and ML bootstrap values (≥95). The colors of the species clades/labels correspond to those used in Figures 4, 7 and 9. [file 12862_2014_223_MOESM1_ESM.jpeg]

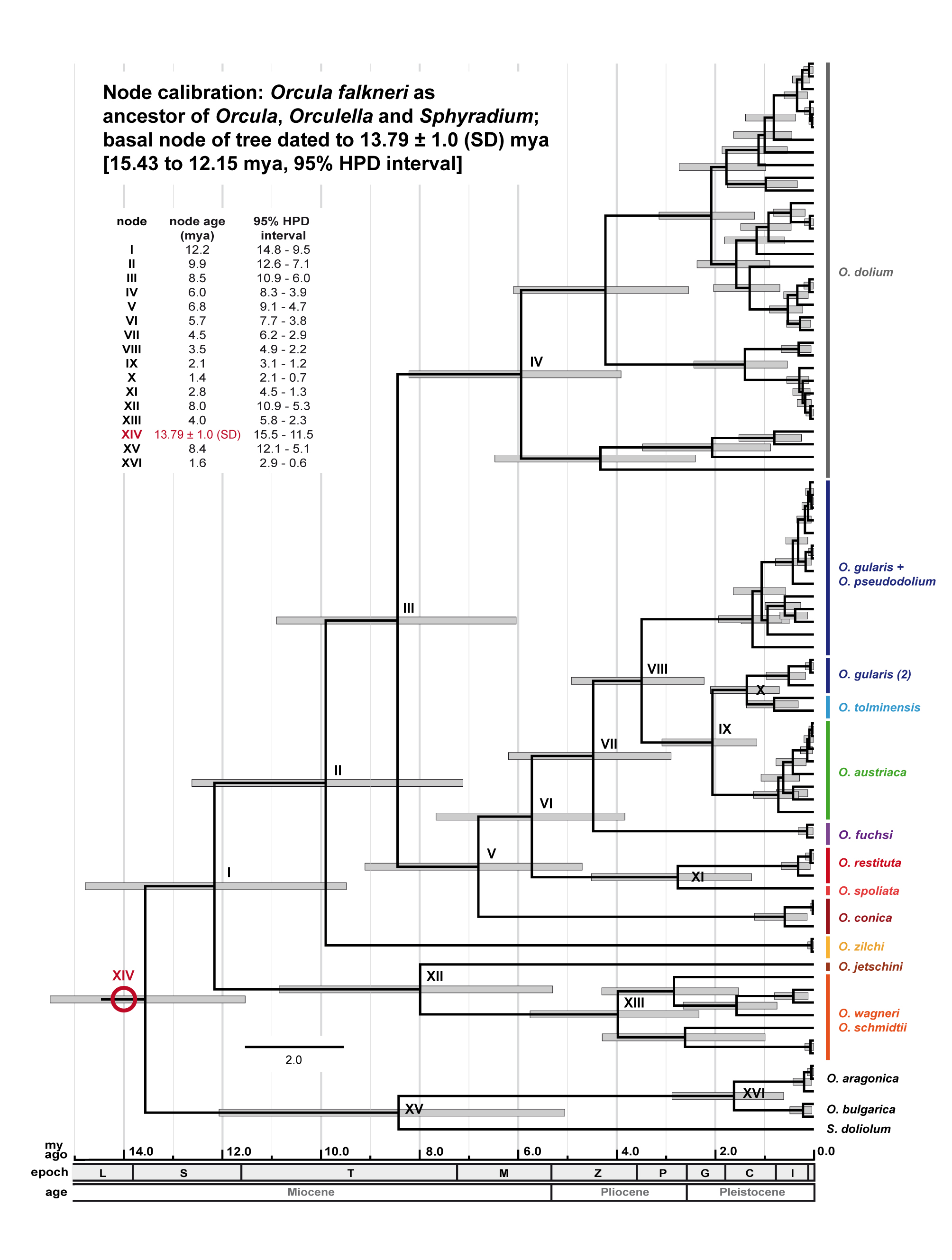

Supplement: Additional file 2: — Linearized molecular clock dated tree (approach 1). Maximum-clade–credibility tree calculated with BEAST, using the concatenated alignments of 12S, 16S and COI. The root of the tree (node XIV) was calibrated to the age of the fossil Nordsieckula falkneri, the presumed most recent common ancestor of Orcula, Sphyradium and Orculella. Node bars indicate the 95% HPD ranges estimated for each node. Mean node ages and 95% HPD ranges are provided for the major splits. A time scale in mya is given below the tree. Abbreviations of the geological epochs: C: Chattian, A: Aquitanian, B: Burdigalian, L: Langhian, S: Serravallian, T: Tortonian, M: Messinian, Z: Zanclean, P: Piacenzian, G: Gelasian and C: Calabrian. [file 12862_2014_223_MOESM2_ESM.jpeg]

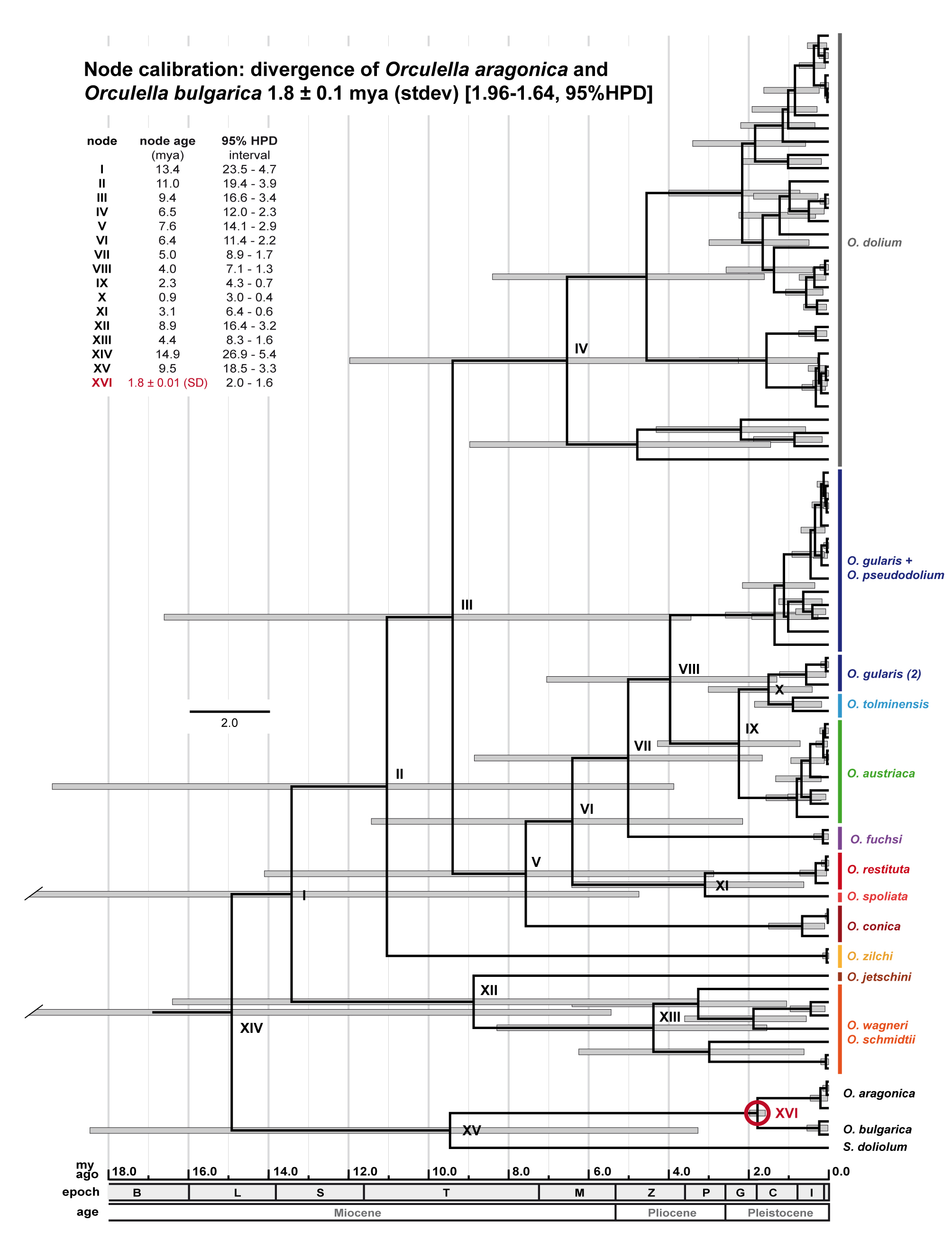

Supplement: Additional file 3: — Linearized molecular clock dated tree (approach 1) . Maximum-clade–credibility tree calculated with BEAST, using the concatenated alignments of 12S, 16S and COI. The divergence date of the outgroups O. bulgarica and O. aragonica (node XVI) was calibrated to the time of the first occurrence of ancestral O. aragonica in the fossil record. Node bars indicate the 95% HPD ranges estimated for each node. Mean node ages and 95% HPD ranges are provided for the major splits. A time scale in mya is given below the tree. Abbreviations of the geological epochs: C: Chattian, A: Aquitanian, B: Burdigalian, L: Langhian, S: Serravallian, T: Tortonian, M: Messinian, Z: Zanclean, P: Piacenzian, G: Gelasian and C: Calabrian. [file 12862_2014_223_MOESM3_ESM.jpeg]

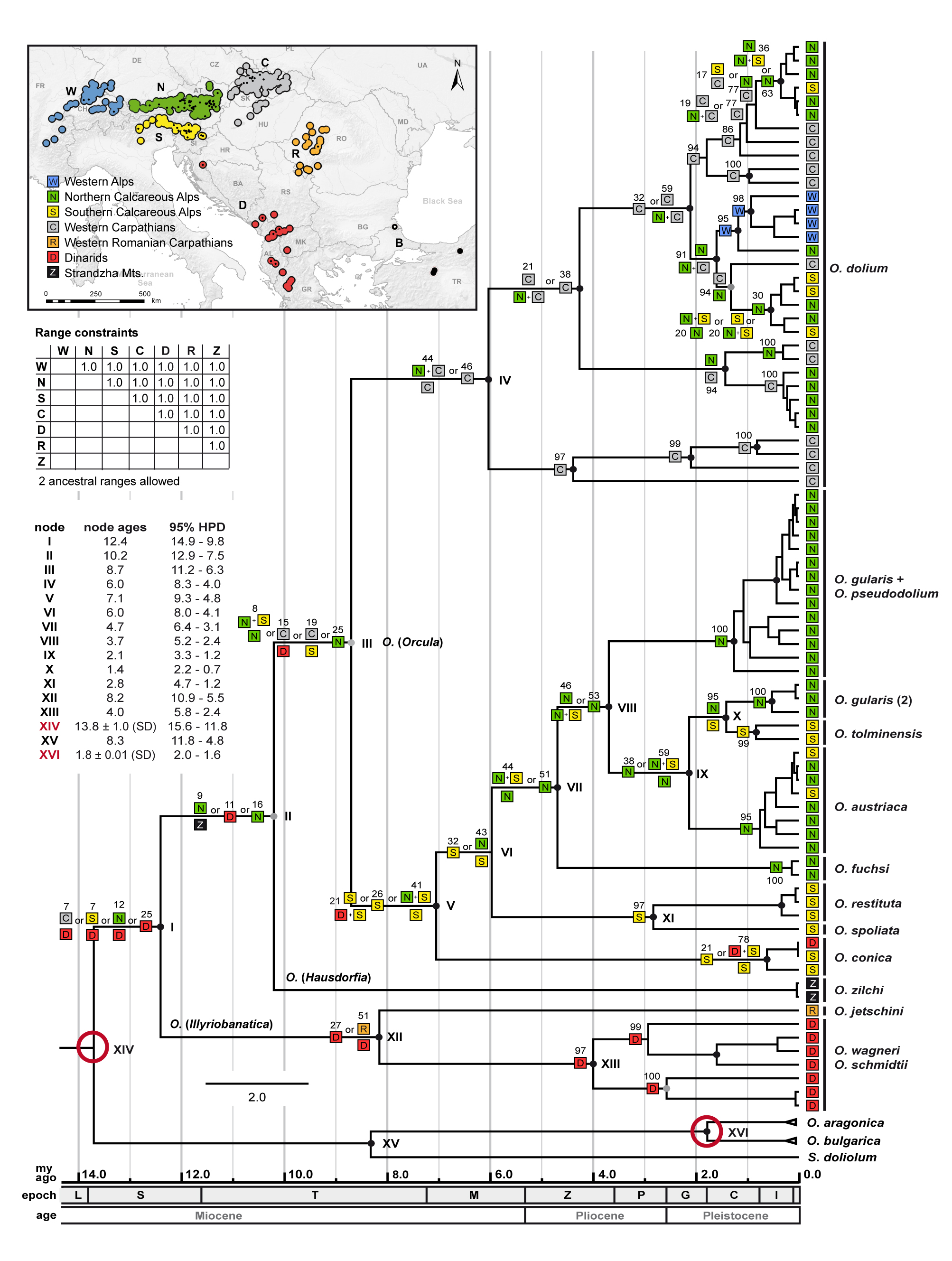

Supplement: Additional file 4: — Reconstruction of the historic geographic ranges (unconstrained model). The linearized molecular clock dated maximum-clade-credibility tree shows the relationships of selected mt lineages (concatenated 16S, 12S and COI sequences). Migration was permitted between all areas and with the same dispersal probabilities. Black dots indicate nodes with high posterior probabilities. The colored symbols at the branch tips indicate the geographic origin of each haplotype. At the cladogenesis events (nodes), all alternative ancestral subdivision/inheritance scenarios with likelihoods of 10% or more are indicated, separated by an “or”, together with the respective likelihoods in%. When scenarios for cladogenesis events involve two ancestral areas, the symbol for the likely ancestral area/-s is/are provided left to each of the two branches. For nodes representing major splits, node ages and 95% posterior HPD intervals are indicated (see Table). A time scale in mya is given below the tree. Abbreviations of the geological epochs: C: Chattian, A: Aquitanian, B: Burdigalian, L: Langhian, S: Serravallian, T: Tortonian, M: Messinian, Z: Zanclean, P: Piacenzian, G: Gelasian and C: Calabrian. [file 12862_2014_223_MOESM4_ESM.jpeg]
