# Supplementary material for: Phylogeography of the land snail genus Orcula (Orculidae, Stylommatophora) with emphasis on the Eastern Alpine taxa: speciation, hybridization and morphological variation
Source: BMC Evol Biol. 2014 Oct 30;14:223. doi: 10.1186/s12862-014-0223-y (PMC4219030; doi:10.1186/s12862-014-0223-y)
Supplement: Additional file 5: — Mean and maximum genetic p -distances in the 12S data set. [file 12862_2014_223_MOESM5_ESM.docx]

**Additional file 1 Mean and maximum genetic *p*-distances (in %) for the *12S* sequences**

|  | **max. dist.** | ***O. dolium*** | ***O. gularis /***  ***O. pseudodolium*** | ***O. gularis* (2)** | ***O. tolminensis*** | ***O. austriaca*** | ***O. fuchsi*** | ***O. restituta*** | ***O. spoliata*** | ***O. conica*** | ***O. schmidtii / O. wagneri*** | ***O. jetschini*** | ***O. zilchi*** |
| --- | --- | --- | --- | --- | --- | --- | --- | --- | --- | --- | --- | --- | --- |
|  |  |  |  |  |  |  |  |  |  |  |  |  |  |
| ***O. dolium*** | **15.6** |  |  |  |  |  |  |  |  |  |  |  |  |
| ***O. gularis / O. pseudodolium*** | **4.3** | 20.4 |  |  |  |  |  |  |  |  |  |  |  |
| ***O. gularis* (2)** | **1.5** | 18.9 | 12.5 |  |  |  |  |  |  |  |  |  |  |
| ***O. tolminensis*** | **3.7** | 19.0 | 13.9 | 5.3 |  |  |  |  |  |  |  |  |  |
| ***O. austriaca*** | **1.5** | 17.3 | 12.2 | 3.8 | 4.9 |  |  |  |  |  |  |  |  |
| ***O. fuchsi*** | **-** | 19.7 | 14.7 | 12.8 | 13.8 | 11.3 |  |  |  |  |  |  |  |
| ***O. restituta*** | **0.9** | 20.5 | 17.5 | 16.6 | 17.0 | 15.2 | 16.0 |  |  |  |  |  |  |
| ***O. spoliata*** | **-** | 17.9 | 15.8 | 15.5 | 15.9 | 14.3 | 15.1 | 8.5 |  |  |  |  |  |
| ***O. conica*** | **2.2** | 18.9 | 17.7 | 16.7 | 15.8 | 15.1 | 16.4 | 13.8 | 13.1 |  |  |  |  |
| ***O. schmidtii / O. wagneri*** | **12.3** | 28.0 | 30.3 | 29.7 | 30.6 | 29.3 | 30.3 | 27.6 | 27.6 | 26.1 |  |  |  |
| ***O. jetschini*** | **0.2** | 28.6 | 29.5 | 27.7 | 27.2 | 27.0 | 30.4 | 26.6 | 25.7 | 25.1 | 25.7 |  |  |
| ***O. zilchi*** | **-** | 27.9 | 29.4 | 26.6 | 27.6 | 26.7 | 28.8 | 30.0 | 27.9 | 26.8 | 31.9 | 28.9 |  |
| ***S. doliolum*** | **-** | **30.8** | **32.4** | **28.7** | **28.8** | **28.2** | **28.8** | **29.1** | **27.3** | **27.6** | **30.6** | **31.1** | **31.6** |
