# Supplementary material for: Phylogeography of the land snail genus Orcula (Orculidae, Stylommatophora) with emphasis on the Eastern Alpine taxa: speciation, hybridization and morphological variation
Source: BMC Evol Biol. 2014 Oct 30;14:223. doi: 10.1186/s12862-014-0223-y (PMC4219030; doi:10.1186/s12862-014-0223-y)
Supplement: Additional file 6: — Mean and maximum genetic p -distances in the 16S data set. [file 12862_2014_223_MOESM6_ESM.docx]

**Additional file 2 Mean and maximum genetic *p*-distances (in %) for the *16S* sequences**

|  | **max. dist.** | ***O. dolium*** | ***O. gularis /***  ***O. pseudodolium*** | ***O. gularis* (2)** | ***O. tolminensis*** | ***O. austriaca*** | ***O. fuchsi*** | ***O. restituta*** | ***O. spoliata*** | ***O. conica*** | ***O. schmidtii / O. wagneri*** | ***O. jetschini*** | ***O. zilchi*** |
| --- | --- | --- | --- | --- | --- | --- | --- | --- | --- | --- | --- | --- | --- |
|  |  |  |  |  |  |  |  |  |  |  |  |  |  |
| ***O. dolium*** | **14.0** |  |  |  |  |  |  |  |  |  |  |  |  |
| ***O. gularis / O. pseudodolium*** | **3.9** | 18.8 |  |  |  |  |  |  |  |  |  |  |  |
| ***O. gularis* (2)** | **1.0** | 16.5 | 11.5 |  |  |  |  |  |  |  |  |  |  |
| ***O. tolminensis*** | **4.8** | 17.6 | 11.9 | 5.0 |  |  |  |  |  |  |  |  |  |
| ***O. austriaca*** | **1.9** | 16.3 | 10.0 | 3.8 | 5.5 |  |  |  |  |  |  |  |  |
| ***O. fuchsi*** | **0.3** | 16.4 | 12.5 | 10.0 | 10.7 | 9.4 |  |  |  |  |  |  |  |
| ***O. restituta*** | **1.3** | 17.9 | 16.5 | 13.6 | 14.7 | 13.2 | 13.7 |  |  |  |  |  |  |
| ***O. spoliata*** | **-** | 18.7 | 16.1 | 14.0 | 15.0 | 12.9 | 14.2 | 8.5 |  |  |  |  |  |
| ***O. conica*** | **1.2** | 17.4 | 16.8 | 14.4 | 15.4 | 14.4 | 13.8 | 14.6 | 15.1 |  |  |  |  |
| ***O. schmidtii / O. wagneri*** | **11.6** | 21.6 | 23.8 | 21.6 | 21.7 | 21.2 | 21.5 | 21.1 | 22.2 | 21.2 |  |  |  |
| ***O. jetschini*** | **-** | 22.5 | 22.4 | 22.4 | 21.6 | 21.3 | 20.0 | 22.2 | 22.7 | 22.6 | 21.0 |  |  |
| ***O. zilchi*** | **-** | 26.0 | 24.2 | 24.5 | 24.9 | 24.1 | 23.5 | 25.6 | 25.6 | 25.0 | 25.4 | 26.4 |  |
| ***S. doliolum*** | **-** | **26.0** | **26.1** | **25.8** | **25.5** | **24.9** | **24.5** | **26.2** | **27.3** | **25.5** | **23.8** | **27.5** | **26.4** |
