# Supplementary material for: Phylogeography of the land snail genus Orcula (Orculidae, Stylommatophora) with emphasis on the Eastern Alpine taxa: speciation, hybridization and morphological variation
Source: BMC Evol Biol. 2014 Oct 30;14:223. doi: 10.1186/s12862-014-0223-y (PMC4219030; doi:10.1186/s12862-014-0223-y)
Supplement: Additional file 7: — Mean and maximum genetic p -distances in the H4 / H3 data set. [file 12862_2014_223_MOESM7_ESM.docx]

**Aditional file 3 Mean and maximum genetic *p*-distances (in %) for the *H4*/*H3* sequences**

|  | ***O. (Orcula)*** | | | ***O. (Illyriobanatica)*** | | | ***O. (Hausdorfia)*** | | |
| --- | --- | --- | --- | --- | --- | --- | --- | --- | --- |
|  |  |  |  |  |  |  |  |  |  |
| **partition** | *H4* | *H3* | ***S.*** | *H4* | *H3* | ***S.*** | *H4* | *H3* | ***S.*** |
| **max. dist.** | **2,7** | **2,9** | **5,9** | **1,2** | **1,2** | **3,9** | **0,4** | **-** | **-** |
| ***O.* (*Orcula*)** |  |  |  |  |  |  |  |  |  |
| ***O.* (*Illyriobanatica*)** | 1,6 | 2,1 | 8,5 |  |  |  |  |  |  |
| ***O.* (*Hausdorfia*)** | 1,4 | 2,4 | 7,2 | 1,6 | 2,1 | 5,1 |  |  |  |
| ***S. doliolum*** | **2,8** | **6,4** | **20** | **2,9** | **5,6** | **17,4** | **2,5** | **5,2** | **17,6** |
